# Glucose-albumin ratio (GAR) as a novel biomarker for predicting postoperative pneumonia (POP) in older adults with hip fractures

**DOI:** 10.1038/s41598-024-60390-2

**Published:** 2024-11-04

**Authors:** Wanyun Tang, Xiaomin Ni, Wei Yao, Wei Wang, Yuhao Li, Qiaomei Lv, Wenbo Ding, Renjian He

**Affiliations:** 1 Department of Orthopedics, Zigong First People’s Hospital, Zigong, China; 2 Department of Orthopedics, Dandong Central Hospital, China Medical University, Dandong, China; 3 Department of Orthopedics, Zigong Fourth People’s Hospital, Zigong, China; 4 Department of Oncology, Dandong Central Hospital, China Medical University, Dandong, China

**Keywords:** Hip fracture, Pneumonia, Glucose-albumin ratio, Biomarker, Predicting, Medical research, Risk factors, Signs and symptoms

## Abstract

Postoperative pneumonia (POP) is a common complication after hip fracture surgery and is associated with increased mortality and other complications in elderly patients. This study aims to evaluate biomarkers, especially the glucose-albumin ratio (GAR), for predicting POP in elderly hip fracture patients. A total of 1279 elderly patients admitted to our hospital with hip fractures were included. We assessed 29 biomarkers and focused on GAR to determine its prognostic and predictive value for POP. Multivariable logistic regression and propensity score-matched analyses were conducted to calculate adjusted odds ratios (ORs) and 95% confidence intervals (CIs) for POP, adjusting for potential confounders. Receiver operating characteristic (ROC) curves were utilized to determine the optimal cut-off of GAR for predicting POP. Among the biomarkers and combinations assessed, GAR demonstrated superior predictive capability for POP in elderly hip fracture patients. ROC analyses showed moderate predictive accuracy of GAR for POP, with an area under the curve of 0.750. Using the optimal cut-off of 0.175, the high GAR group was significantly associated with increased odds of POP (adjusted OR 2.14, 95%, CI 1.50–3.05). These associations remained significant after propensity score matching and subgroup analyses. Dose–response relationships between GAR and POP were observed. In conclusion, GAR may be a promising biomarker to predict POP risk in elderly hip fracture patients. Further studies are warranted to validate its clinical utility. However, this study has certain limitations, including its retrospective design, potential for selection bias due to the exclusion criteria, and the single-center nature of the study, which should be addressed in future prospective, multicenter studies.

## Introduction

Hip fractures are a significant health concern within the geriatric population^1^. By 2050, over 6 million hip fracture patients worldwide are projected^1,2^. One-year mortality rates for hip fracture patients range from 14 to 58%^3^. In the United States alone, over 300,000 older adults are hospitalized for hip fractures annually, with an estimated healthcare cost exceeding $10 billion^4^. The incidence of hip fractures is expected to rise as the global population ages, posing a substantial burden on healthcare systems worldwide^5^.

Early surgery is the primary treatment to reduce mortality, postoperative complications remain widespread^6^. Postoperative pneumonia (POP) is one of the most frequent complications, with reported incidence rates ranging from 3 to 15% in recent studies^7–9^. POP often increases hospital stays by 7–9 days, healthcare costs by $40,000–$50,000 per patient, and mortality risk 2–4 times compared to patients without POP^10–12^. Identifying high-risk individuals enables targeted intervention and improves care quality.

Currently, clinical practices for assessing POP risk and managing hip fracture patients postoperatively primarily rely on patient characteristics, such as age, comorbidities, and functional status^5^. However, these factors alone may not accurately predict an individual's risk of developing POP. Additionally, postoperative management strategies, including early mobilization, respiratory therapy, and prophylactic antibiotics, are implemented to mitigate POP risk, but their effectiveness varies among patients^13^. There is a need for more robust risk assessment tools and personalized management approaches to optimize outcomes for hip fracture patients.

Blood biomarkers have become an ideal indicator for evaluating the inflammatory status of perioperative patients due to their ease of acquisition, economy, and practicality^14,15^. Prior studies have investigated various biomarkers for POP prediction. For instance, one study investigated the potential of procalcitonin (PCT) and C-reactive protein (CRP) as early indicators for diagnosing postoperative hospital-acquired pneumonia (HAP) subsequent to abdominal surgery^16^. Another study focused on evaluating the relationship between the neutrophil–lymphocyte ratio (NLR), platelet-lymphocyte ratio (PLR), and systemic immune inflammation index (SII) and the occurrence of POP among elderly patients who had undergone hip fracture surgeries^17^. While CRP, PCT, NLR, PLR, and SII have shown associations with pneumonia in specific surgical populations, these general inflammatory biomarkers lack specificity for pneumonia pathogenesis. Many of the previously studied biomarkers for predicting POP, such as CRP and PCT, lack specificity as they are non-specific inflammatory markers, making it challenging to differentiate POP from other postoperative complications or infections^18^. Furthermore, some of these biomarkers have shown inconsistent or suboptimal performance in predicting POP, particularly in the geriatric population with hip fractures, which often presents with comorbidities and altered physiological states. Additionally, the optimal timing for measuring certain biomarkers concerning the development of POP remains unclear, limiting their practical utility in clinical settings. Moreover, some biomarkers require specialized laboratory tests or equipment, which may not be readily available or cost-effective in all healthcare settings, limiting their accessibility and practical application in risk stratification and management of POP in this vulnerable patient population.

Leveraging readily available and inexpensive laboratory tests, making GAR a cost-effective and accessible biomarker. The glucose-albumin ratio (GAR) has recently emerged as a prognostic indicator across postoperative cohorts but has not been extensively studied for POP prediction after hip fractures^19^. GAR provides a ratio of serum glucose to albumin levels. Elevated GAR indicates increased serum glucose and decreased serum albumin, reflecting factors implicated in infection risks like poor nutrition, inflammation, and hypermetabolism. Furthermore, hyperglycemia suppresses immune function, while hypoalbuminemia reduces the binding and transportation of medications, potentially increasing pneumonia risk^20,21^. This strong biological rationale suggests GAR may specifically predict POP risk in vulnerable elderly hip fracture patients. Additionally, we also compared other various biomarker combinations.

This study aims to investigate the relationship between a novel biomarker (GAR) and POP incidence after hip fracture surgery. This study may provide evidence to improve risk assessment and outcomes for this high-risk patient population, aiding targeted pneumonia prevention.

## Methods

### Study design and participants

This retrospective cohort study was approved by the institutional review board at our hospital (No. DDZX-20231001). Data was collected on 2403 elderly patients aged 60 or above who underwent hip fracture surgery at a local hospital from August 2011 to September 2023. The STROBE guidelines for observational studies were followed. Inclusion criteria: (1) Aged 60 or above; (2) Hip fracture diagnosis confirmed by X-ray or CT imaging; (3) Hip fracture confirmed during surgery.

To minimize potential confounding factors and biases that could influence the interpretation of our findings, we implemented exclusion criteria to establish a well-defined study population focused on acute hip fracture patients without pre-existing respiratory complications, multiple fractures, pathological fractures, chronic or open fractures, or incomplete data. Exclusion criteria: (1) Lack of surgical treatment; (2) Patients with a history of recent pneumonia; (3) Multiple fractures; (4) Pathological fractures; (5) Pre-existing or open fractures; and (6) Incomplete or unavailable data.

The exclusion of these patients was based on a set of predefined criteria, which were implemented to ensure a well-defined and homogeneous study population. The specific criteria were as follows: (1) Lack of surgical treatment: Our study focused on postoperative pneumonia in patients who underwent surgical intervention for hip fractures. Patients who were managed conservatively without surgery were excluded to maintain consistency with the research objectives. (2) Patients with a history of recent pneumonia: Patients with a history of pneumonia within a specified time frame (1 month) prior to the hip fracture surgery were excluded to eliminate potential confounding effects of pre-existing respiratory conditions on the postoperative pneumonia risk. (3) Multiple fractures: Patients with multiple fractures, in addition to the hip fracture, were excluded to minimize the potential confounding effects of additional injuries or complications on the risk of postoperative pneumonia and the interpretation of our results. (4)Pathological fractures: Fractures caused by underlying conditions, such as bone tumors or metastases, were classified as pathological fractures and excluded to maintain a more homogeneous study population with a similar underlying etiology for their fractures. (5) Pre-existing or open fractures: Patients with pre-existing or open fractures were excluded to ensure that the study focused solely on acute hip fractures and to avoid potential confounding factors associated with chronic or complicated fractures. (6) Incomplete or unavailable data: Patients with incomplete or missing data for key variables required for the analysis, such as biomarker levels or relevant clinical information, were excluded to maintain the integrity and completeness of the dataset for reliable statistical analyses.

After applying the exclusion criteria, 1124 patients were removed, leaving a final retrospective cohort of 1279 patients for comprehensive analysis. Participant selection is illustrated in a flowchart (Supplementary eFigure 1).

### The sample sizes

The events per variable (EPV) method is a rule of thumb used to assess whether the sample size is sufficient for a logistic regression model^22^. The method states that the model estimates are reliable only if there are around 10 events for each variable in the model. EPV calculation formula: EPV = Number of events / Number of variables.

EPV cutoff values and their interpretation^23^: EPV ≥ 10: The sample size is usually sufficient. This means that the model estimates are likely to be reliable, with good precision and low bias. 5 ≤ EPV < 10: The sample size may be insufficient. This means that the model estimates may be less precise and more biased. The researcher should carefully consider the results and may need to further investigate the model's performance. EPV < 5: The sample size is very likely insufficient. This means that the model estimates are likely to be unreliable and should be interpreted with caution. The researcher should increase the sample size or reduce the number of variables in the model. The EPV value of our study is calculated as follows: EPV = 1279* 0.092 / 13 = 9. The EPV value is around 10, the sample size of our study is sufficient.

### Data collection

Demographic and clinical data were extracted from the hospital's electronic medical records. The following preoperative information was collected: (1) demographics: age, gender, smoking status, alcohol use; (2) comorbidities: hypertension, diabetes, COPD, cardiovascular disease, stroke, dementia, intracerebral hemorrhage, chronic liver disease, chronic kidney disease, rheumatoid arthritis; (3) clinical characteristics: fracture type, surgery type, bedridden time, operative blood loss, surgery time, transfusion, postoperative ICU admission, American Society of Anesthesiologists (ASA).

To ensure data completeness and quality, we implemented strict criteria for patient inclusion in the analysis. Patients with missing or incomplete laboratory test results (including glucose and albumin levels), radiological data (CT scans or X-rays), or critical clinical information (such as medical history, comorbidities, or postoperative complications) were excluded from the final analysis cohort. Only patients with complete records for all relevant variables were included to minimize potential biases due to missing data.

Additionally, serum concentrations of 29 biomarkers were analyzed, with a focus on glucose and albumin levels. For patients with multiple preoperative measurements, the values closest to admission were used. The Glucose-Albumin Ratio (GAR) was calculated for each sample by dividing the glucose level (mg/dL) by the albumin level (g/dL).

### Outcome

The primary outcome was postoperative pneumonia (POP) within 30 days after surgery. A 30-day postoperative period was chosen to define pneumonia as the primary outcome to align with common practice in high-quality studies, capture both early and delayed cases potentially influenced by the biomarkers under investigation and maintain a feasible follow-up duration that ensures data completeness while minimizing confounding factors unrelated to the surgical procedure itself. POP was defined based on diagnostic criteria from the American College of Chest Physicians (ACCP) and European Respiratory Society (ERS)^24^.

A POP diagnosis required meeting one or more of the following criteria: (1) the emergence or exacerbation of respiratory symptoms, such as cough and purulent discharge; (2) a body temperature exceeding 38 °C or falling below 36.0 °C; (3) the identification of lung consolidation or the presence of crackles during a physical examination; (4) an abnormal white blood cell count indicative of either leukocytosis (> 10 × 109/L) or leukopenia (< 4 × 109/L); (5) the confirmation of relevant pathogens through microbial culture in sputum or blood samples. Two blinded respiratory physicians identified POP through individual chart reviews. Any conflicts were resolved by a third respiratory physician.

### Statistical analysis

Baseline demographic and clinical characteristics were summarized using descriptive statistics. Continuous variables were reported as mean and standard deviation. Categorical variables were reported as frequency and percentage. We use the specific imputation method used to handle minor missing data points.

The receiver operating characteristic (ROC) curve analyzed the diagnostic efficacy of GAR. The area under the ROC curve (AUC) compared diagnostic performance. Youden's index maximization determined the optimal GAR cutoff. Sensitivity, specificity, positive predictive value (PPV), negative predictive value (NPV), and 95% confidence intervals (CIs) assessed GAR's predictive ability for POP. GAR levels were categorized using the optimal cutoff. Multivariate logistic regression analysis, adjusted for confounders, examined the association between GAR and POP. Variables with *p* < 0.05 in univariate regression were included in the multivariate model.

To minimize potential confounding effects and covariate adjustments, we employed propensity score matching (PSM) with the nearest neighbor algorithm, matching covariates in a 1:1 ratio between groups. A caliper width of 0.25 standard deviations (SD) was used. Group characteristics were compared using standardized mean differences (SMDs). Furthermore, we stratified patients into quartiles (Q1, Q2, Q3, and Q4) based on GAR levels to precisely assess the GAR-POP dose–response relationship.

In the PSM cohort, we conducted the subgroup analysis to further explore GAR's diagnostic utility. We stratified the PSM cohort into multiple subgroups based on all covariates and conducted univariate logistic regression analysis to determine the odds ratio (OR) and 95% confidence interval (CI) for high GAR's association with POP.

Data analysis was conducted using SPSS version 26.0 (IBM Corp., Armonk, New York, USA) for statistical analysis and R version 4.0.3 (R Foundation for Statistical Computing, Vienna, Austria).

### Ethics approval and consent to participate

This study was approved by the Ethics Committee of Dandong Central Hospital (No. DDZX-20231001) and conducted by the ethical principles outlined in the Helsinki Declaration of 1964 and its subsequent amendments. The Institutional Review Board of Dandong Central Hospital waived the requirement for informed consent for the cohort study to reduce potential duplication of effort.

## Results

**Figure 1:**
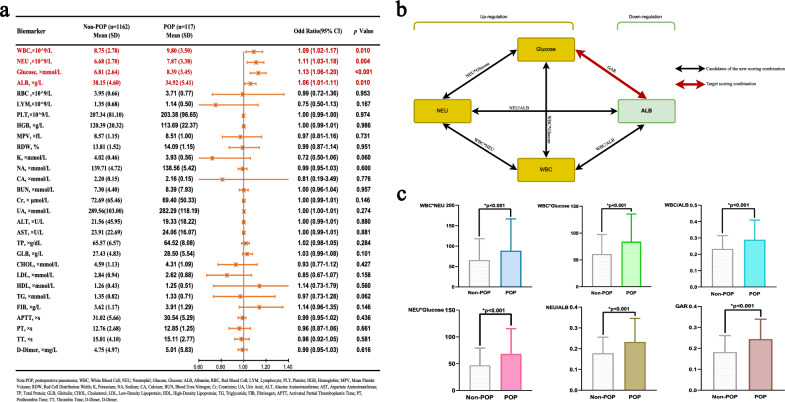
(**a**) Baseline data and multivariate regression analyses of each laboratory factor for POP. (**b**) Schematic chart for the combination of laboratory biomarkers in this study. (**c**) The bar graph displays the distribution of new combinations of four biomarkers between the non-POP group and the POP group.

The study population included 1279 patients undergoing surgery for hip fracture, of whom 117 (9.1%) developed POP. Patients who developed POP were significantly older than those without POP (mean 80.5 vs 74.1 years, *p* < 0.001). POP patients also had a higher prevalence of comorbidities including COPD (41.0% vs 8.8%, *p* < 0.001), cardiovascular disease, stroke, dementia, and chronic liver and kidney disease (all *p* < 0.05). POP patients were more likely to have been readmitted (41.9% vs 27.8%, *p* = 0.001), have longer postoperative bedridden time (mean 7.9 vs 5.7 days, *p* < 0.001), require postoperative ICU admission (14.5% vs 4.2%, *p* < 0.001), and have higher ASA scores of III-V (76.9% vs 53.5%, p < 0.001) (Table 1). In addition, the WBC (*p* = 0.010), NEU (*p* = 0.004), glucose (*p* < 0.001) and ALB (*p* = 0.010) were all significantly elevated in the POP group (Fig. 1a). Figure 1b,c compare the Non-POP and POP groups on the new combinations of four biomarkers, respectively.

**Table 1:** Baseline demographic characteristics of patients with and without predicting POP.

	Total (n = 1279)	Without POP (n = 1162)	With POP (= 117)	*p*-value
Demographic				
Age, × years (Mean, SD)	74.70 (9.55)	74.12 (9.49)	80.52 (8.05)	< 0.001
Female gender (n, %)	771 (60.3)	700 (60.2)	71 (60.7)	0.926
Smoking (n, %)	218 (17.0)	197 (17.0)	21 (17.9)	0.785
Alcohol (n, %)	148 (11.6)	134 (11.5)	14 (12.0)	0.889
Comorbidities				
Hypertension (n, %)	641 (50.1)	573 (49.3)	68 (58.1)	0.069
Diabetes (n, %)	297 (23.2)	263 (22.6)	34 (29.1)	0.117
COPD (n, %)	150 (11.7)	102 (8.8)	48 (41.0)	< 0.001
Cardiovascular (n, %)	394 (30.8)	343 (29.5)	51 (43.6)	0.002
Stroke (n, %)	332 (26.0)	282 (24.3)	50 (42.7)	< 0.001
Dementia (n, %)	48 (3.8)	39 (3.4)	9 (7.7)	0.019
Intracerebral hemorrhage (n, %)	70 (5.5)	63 (5.4)	7 (6.0)	0.799
Chronic liver disease (n, %)	58 (4.5)	48 (4.1)	10 (8.5)	0.029
Chronic kidney disease (n, %)	65 (5.1)	53 (4.6)	12 (10.3)	0.008
Rheumatoid arthritis (n, %)	22 (1.7)	22 (1.9)	0 (0)	0.133
Readmission (n, %)	372 (29.1)	323 (27.8)	49 (41.9)	0.001
Fracture type				
Femoral neck fracture (n, %)	684 (53.5)	639 (55.0)	45 (38.5)	< 0.001
Intertrochanteric fracture (n, %)	521 (40.7)	452 (38.9)	69 (58.9)	
Subtrochanteric fracture (n, %)	74 (5.8)	71 (6.1)	3 (2.6)	
Surgery type				
Total hip arthroplasty (n, %)	162 (12.7)	151 (13.0)	11 (9.4)	0.004
Hemiarthroplasty (n, %)	322 (25.2)	293 (25.2)	29 (24.8)	
Intramedullary nail (n, %)	416 (32.5)	364 (31.3)	52 (44.4)	
Plate/screw (n, %)	170 (13.3)	152 (13.1)	18 (15.4)	
Multiple screws (n, %)	209 (16.3)	202 (17.4)	7 (6.0)	
Bedridden time, × days (Mean, SD)	5.89 (4.02)	5.69 (3.87)	7.85 (4.93)	< 0.001
Operative blood loss, × ml (Mean, SD)	175.17 (152.54)	174.06 (154.37)	186.26 (132.98)	0.410
Surgery time, × hours (Mean, SD)	1.66 (0.80)	1.66 (0.81)	1.71 (0.73)	0.493
Transfusion (n, %)	210 (16.4)	189 (16.3)	21 (17.9)	0.639
Postoperative ICU (n, %)	66 (5.2)	49 (4.2)	17 (14.5)	< 0.001
ASA				
III–V (n, %)	712 (55.7)	622 (53.5)	90 (76.9)	< 0.001
I–II (n, %)	567 (44.3)	540 (46.5)	27 (23.1)	

Data are presented as median (Mean, SD) and n (%).

*POP* postoperative pneumonia, *COPD* chronic obstructive pulmonary disease, *ASA* American Society of Anesthesiologists score.

Variables with *p* < 0.05 in univariate regression were included in the multivariate model. In the multivariate analysis, Age, COPD, Cardiovascular, Stroke, Dementia, Chronic liver disease, Chronic kidney disease, Readmission, Fracture type, Bedridden time, Postoperative ICU, ASA, GAR ≥ 0.175 mmol/L were included. In multivariate analysis adjusting for potential confounders, Age (OR 1.04), COPD (OR 5.50), Stroke (OR 1.71), Bedridden time (OR 1.06), Postoperative ICU stay (OR 2.52), and GAR ≥ 0.175 (OR 3.91) remained independent risk factors for POP (Table 2).

**Table 2:** Univariate and multivariate regression analyses of risk factors for POP.

Characteristics	Univariate			Multivariate		
	OR	95%CI	*p*-value	OR	95%CI	*p*-value
Age	1.08	1.05–1.10	< 0.001	1.04	1.01–1.07	0.003
Female gender	0.93	0.67–1.45	0.926	< NA >	< NA >	< NA >
Smoking	1.07	0.65–1.76	0.785	< NA >	< NA >	< NA >
Alcohol	1.04	0.58–1.88	0.889	< NA >	< NA >	< NA >
Hypertension	1.43	0.97–2.10	0.070	< NA >	< NA >	< NA >
Diabetes	1.40	0.92–2.14	0.118	< NA >	< NA >	< NA >
COPD	7.23	4.75–11.01	< 0.001	5.50	3.42–8.85	< 0.001
Cardiovascular	1.85	1.25–2.72	0.002	0.86	0.54–1.35	0.505
Stroke	2.34	1.58–3.44	< 0.001	1.71	1.09–2.67	0.020
Dementia	2.40	1.13–5.09	0.022	1.65	0.69–3.96	0.264
Intracerebral hemorrhage	1.11	0.50–2.48	0.799	< NA >	< NA >	< NA >
Chronic liver disease	2.17	1.07–4.41	0.032	1.48	0.64–3.44	0.360
Chronic kidney disease	2.39	1.24–4.62	0.009	1.62	0.73–3.60	0.238
Rheumatoid arthritis	NA	NA	NA	< NA >	< NA >	< NA >
Readmission	1.87	1.27–2.76	0.002	1.32	0.84–2.09	0.230
Fracture type	0.71	0.53–0.96	0.027	0.97	0.67–1.40	0.870
Surgery type	0.92	0.79–1.07	0.285	< NA >	< NA >	< NA >
Bedridden time	1.10	1.06–1.14	< 0.001	1.06	1.01–1.10	0.009
Operative blood loss	1.00	1.00–1.00	0.410	< NA >	< NA >	< NA >
Surgery time	1.08	0.87–1.35	0.493	< NA >	< NA >	< NA >
Transfusion	1.13	0.69–1.85	0.640	< NA >	< NA >	< NA >
Postoperative ICU	3.86	2.14–6.96	< 0.001	2.52	1.28–4.97	0.008
ASA	2.89	1.85–4.52	< 0.001	1.29	0.77–2.14	0.330
GAR ≥ 0.175 mmol/L	6.29	3.65–10.00	< 0.001	3.91	2.36–6.48	< 0.001

*p*-value: *p* < 0.05 indicates that the risk factor is statistically significantly associated with the POP outcome. In the multivariate analysis, Age, COPD, Cardiovascular, Stroke, Dementia, Chronic liver disease, Chronic kidney disease, Readmission, Fracture type, Bedridden time, Postoperative ICU, ASA, GAR ≥ 0.175 mmol/L were included. In multivariate analysis adjusting for potential confounders, Age (1.04), COPD (OR 5.50), Stroke (OR 1.71), Bedridden time (OR 1.06), Postoperative ICU (OR 2.52), and GAR ≥ 0.175 (OR 3.91) remained independent risk factors for POP.

*POP* postoperative pneumonia, *COPD* chronic obstructive pulmonary disease, *ASA* American Society of Anesthesiologists score, *GAR* glucose to albumin ratio.

According to the optimal cutoff values of WBC, NEU, glucose and ALB, detailed results of the multivariable regression analysis can be found in supplementary eTables 1–4. Elevated levels of WBC (OR = 1.09, 95% CI 1.02–1.17), NEU (OR = 1.11, 95% CI 1.03–1.18), glucose (OR = 1.13, 95% CI 1.06–1.20) and ALB (OR = 1.06, 95% CI 1.01–1.11) exhibited a significant correlation with POP.

**Figure 2:**
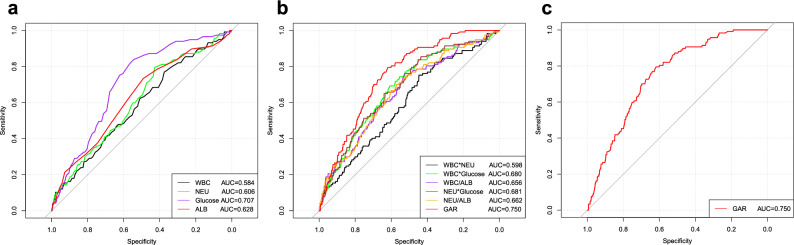
ROC curves analysis to evaluate the predictive value of each combination for POP in patients with hip fractures. (**a**) ROC curves analysis for single laboratory factor. (**b**) ROC curves analysis for each new combination of four biomarkers. (**c**) ROC curves analysis for GAR.

The diagnostic performance of individual and combined blood-based biomarkers for predicting POP was examined by ROC curve analysis (Table 3 and Fig. 2). The glucose-albumin ratio (GAR) showed the best diagnostic accuracy among all markers, with an AUC of 0.750 (95% CI 0.710–0.790), a cutoff point of 0.175, sensitivity of 79.5%, specificity of 61.9%, and accuracy of 63.5% (Table 3 and Fig. 2c). Among the individual biomarkers, the AUC values for WBC, NEU, glucose, and ALB in predicting POP were 0.584, 0.606, 0.628, and 0.707, respectively (Fig. 2a). Combinations of biomarkers, the areas under the ROC curve for WBC*NEU, WBC*Glucose, WBC/ALB, NEU*Glucose, and NEU/ALB were 0.598, 0.680, 0.656, 0.681, and 0.662 (Fig. 2b).In addition, we also calculated the cutoff values of biomarkers and their combination (supplementary eFigure 2). The selection of the GAR cutoff value of 0.175 was based on a combination of statistical analysis and clinical considerations. First, we performed a receiver operating characteristic (ROC) curve analysis to evaluate the diagnostic performance of GAR in predicting postoperative pneumonia (POP). The area under the ROC curve (AUC) was calculated, and the Youden index was used to identify the optimal cutoff value that maximized both sensitivity and specificity. The GAR value of 0.175 yielded the highest Youden index, indicating the optimal trade-off between sensitivity and specificity for predicting POP in our cohort (supplementary eFigure 2).

**Table 3:** Assessment of the characteristic parameters of each biomarker for predicting POP.

Blood-based biomarkers	Cutoff point	AUC (95% CI)	ACC (%, 95% CI)	SEN (%, 95% CI)	SPE (%, 95% CI)	PPV (%, 95% CI)	NPV (%, 95% CI)
Single biomarkers							
WBC	7.650	0.584 (0.530–0.638)	41.1 (41.1–41.2)	76.9 (69.3–84.6)	37.5 (34.7–40.3)	11.0 (8.9–12.2)	94.2 (92.2–96.3)
NEU	5.890	0.606 (0.554–0.659)	46.2 (46.2–46.2)	79.5 (72.2–86.8)	42.9 (40.0–45.7)	12.3 (9.9–14.6)	95.4 (93.6–97.2)
Glucose	6.150	0.628 (0.572–0.682)	51.3 (51.3–51.3)	73.5 (65.5–81.5)	49.1 (46.2–51.9)	12.7 (10.2–15.2)	94.8 (93.1–96.6)
ALB	38.070	0.707 (0.663–0.751)	57.3 (57.3–57.3)	83.8 (77.7–90.4)	54.6 (51.8–57.5)	15.7 (12.8–18.5)	97.1 (95.8–98.4)
Combinations							
WBC*NEU	47.670	0.598 (0.545–0.652)	47.5 (47.4–47.5)	75.2 (67.4–83.0)	44.7 (41.8–47.5)	12.0 (9.7–14.4)	94.7 (92.8–96.6)
WBC*Glucose	58.400	0.680 (0.630–0.730)	61.6 (61.6–61.6)	69.2 (60.9–77.6)	60.8 (58.0–63.6)	15.1 (12.1–18.1)	95.2 (93.6–96.7)
WBC/ALB	0.225	0.656 (0.604–0.709)	54.8 (54.8–54.8)	76.1 (68.3–83.8)	52.7 (49.8–55.5)	13.9 (11.2–16.6)	95.6 (94.0–97.2)
NEU*Glucose	37.215	0.681 (0.631–0.730)	47.4 (47.3–47.4)	85.5 (79.1–91.9)	43.5 (40.7–46.4)	13.2 (10.8–15.6)	96.7 (95.2–98.3)
NEU/ALB	0.179	0.662 (0.611–0.714)	58.2 (58.2–58.3)	70.1 (61.8–78.4)	57.1 (54.2–59.9)	14.1 (11.3–16.9)	95.0 (93.4–96.6)
GAR	0.175	0.750 (0.710–0.790)	63.5 (63.5–63.5)	79.5 (72.2–86.8)	61.9 (59.1–64.7)	17.4 (14.1–20.6)	96.8 (95.5–98.0)

*AUC* area under curve, *ACC* accuracy, *SEN* sensitivity, *SPE* specificity, *PPV* positive predictive value, *NPV* negative predictive value, *WBC* White Blood Cell, *NEU* neutrophil, *ALB* albumin, *GAR* glucose to albumin ratio.

Higher GAR was associated with significantly increased odds of POP (Table 4). In unadjusted analysis, a GAR ≥ 0.175 was associated with 6.29 times higher odds of events compared to a GAR < 0.175 (95% CI 3.65–10.00, *p* < 0.001). After adjustment for potential confounders in multivariable regression, the strength of the association was attenuated but remained significant (adjusted OR 3.91, 95% CI 2.36–6.48, *p* < 0.001). The association persisted after PSM analysis (OR 2.14, 95% CI 1.50–3.05, *p* < 0.001). The baseline data characteristics of different groups of the GAR cutoffs and the GAR quartiles before and after PSM are shown in eTable 5–9. After matching on the propensity score, there were no statistically significant differences between the POP and non-POP groups on any of the measured baseline covariates, indicating the matching process sufficiently balanced the groups.

**Table 4:** Unadjusted and adjusted associations between POP and GAR based on different cutoff values.

Hematologic parameters	Categories	Glucose-albumin ratio	Events, n (%)	Unadjusted OR (95% CI)	*p	Multivariable regression adjusted OR (95% CI)	*p	PSM adjusted OR (95% CI)	*p
GAR	Cutoff	< 0.175	24/743 (3.2)	1 [Reference]	< 0.001	1 [Reference]	< 0.001	1 [Reference]	< 0.001
		≥ 0.175	93/536/ (17.3)	6.29 (3.65–10.00)		3.91 (2.36–6.48)		2.14 (1.50–3.05)	
	Quartile	Q1 (< 0.137)	3/320 (0.9)	1 [Reference]	NA	1 [Reference]	NA	1 [Reference]	NA
		Q2 (0.137–0.164)	16/320 (5.0)	5.56 (1.60–19.28)	0.007	3.08 (0.76–12.42)	0.114	1.20 (0.37–3.89)	0.764
		Q3 (0.164–0.206)	36/320 (11.2)	13.39 (4.08–43.97)	< 0.001	9.51 (2.75–32.84)	< 0.001	2.12 (1.22–3.68)	0.008
		Q4 (≥ 0.206)	62/319 (19.4)	25.49 (7.91–82.16)	< 0.001	10.57 (3.02–3 6.97)	< 0.001	3.55 (1.93–6.50)	< 0.001

*GAR* glucose to albumin ratio, *NA* not available, *OR* odds ratio, *PSM* propensity scores matching.

*p for trend.

**Figure 3:**
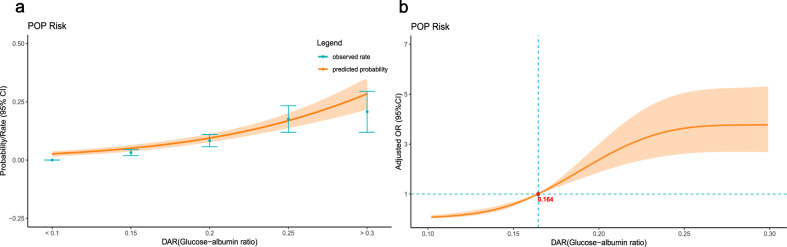
Relationship between GAR level and POP in patients with hip fracture. (**A**) Predicted probabilities and the observed rate of POP; (**B**) Adjusted odd ratios (OR) and corresponding 95% confidence intervals (CI) are presented for each 0.05 deviation from the reference value of 0.164 mmol/L.

The results demonstrate a dose-dependent relationship between preoperative GAR and POP risk in elderly hip fracture patients (Table 4). When examined by quartiles, higher GAR quartiles (Q3 and Q4) were associated with stepwise increases in the odds of events compared to the lowest quartile (Q1) in adjusted analyses. We calculated the predicted probability and observed incidence rates of POP by considering preoperative GAR levels. Our analysis revealed a positive correlation between increasing preoperative GAR levels and the likelihood of developing POP, as illustrated in Fig. 3a. Notably, patients with elevated preoperative GAR levels exhibited a higher risk of developing POP compared to those with a GAR level of 0.164 (used as a reference). Moreover, when examined as a continuous variable, higher GAR levels remained associated with an elevated risk of pneumonia, as shown in Fig. 3b.

We performed supplementary analyses to assess the potential interplay between GAR and various factors, as detailed in eFigure 3. Notably, we detected a significant interaction between GAR and hypertension (interaction *p* < 0.05), suggesting that the impact of GAR on the susceptibility to POP may exhibit variations contingent on the presence of hypertension. Nevertheless, it is important to note that no substantial effect modification of GAR and POP was observed with respect to other variables.

Additionally, the study examined the association between GAR and postoperative pneumonia after hip fracture surgery in different fractures and surgery. Patients with femoral neck fractures had 5.52 times higher odds of developing pneumonia postoperatively, with a 95% confidence interval of 2.21–13.81 and a p-value less than 0.001. Those with intertrochanteric fractures had 2.85 times higher odds, with a 95% CI of 1.42–5.74 and p = 0.003. Regarding surgery type, hemiarthroplasty conferred 3.82 times higher odds, with a 95% CI of 1.43–10.22 and p = 0.008. Intramedullary nailing was associated with 2.25 times higher odds (95% CI 1.06–4.80, *p* = 0.036), while plate/screw fixation had notably elevated odds of 10.21 (95% CI 1.26–82.95, *p* = 0.030). However, subtrochanteric fractures, total hip arthroplasty, and multiple screw fixation did not demonstrate statistically significant associations with increased postoperative pneumonia risk (eFigure 3).

## Discussion

In this retrospective cohort study, we explored for the first time the relationship between GAR and POP in elderly patients with hip fractures. Our results indicate that there is a positive correlation between GAR and the incidence of POP, even after multiple regression adjustments and PSM adjustments. Moreover, our results reveal a statistically significant correlation between elevated GAR levels, exceeding the specified threshold of 0.175, and an elevated risk of developing POP. Among all the biomarker combinations investigated, GAR exhibited the strongest predictive capability for POP in elderly patients with hip fractures. This study makes a novel contribution by introducing GAR as a potent preoperative predictor of POP susceptibility in this high-risk population.

Our research findings are consistent with many previous studies, which demonstrate associations between low preoperative serum albumin levels and increased postoperative pulmonary complications, including pneumonia. The study conducted by Danny Lee found that in patients undergoing Total Shoulder Arthroplasty (TSA), low serum albumin levels are a significant risk factor for POP (OR 9.678, *p* = 0.031)^25^. Yakang Wang et al.^25^ found that in a elderly population undergoing surgery for femoral neck fractures, preoperative hypoalbuminemia was identified as a significant predictor of POP, (OR: 5.187, 95%CI: 2.561–10.506, *p* < 0.001). In a study with 3,147 patients^26^, Yunxu Tian et al. found preoperative hypoalbuminemia was independently and significantly associated with an increased risk of developing POP in elderly patients undergoing hip fracture surgeries (OR: 6.18, 95% CI, 3.15–11.98, *p* < 0.001). A systematic review and meta-analysis of 24 studies involving 288,819 participants demonstrated that low serum albumin is an important risk factor for POP in elderly patients undergoing hip fracture surgery, with a pooled odds ratio of 2.34 (95% CI 0.82–6.73)^27^. Multiple mechanisms may underlie this relationship between low serum albumin and heightened susceptibility to POP.

Firstly, as an endogenous antioxidant and modulator of immune function, hypoalbuminemia can impair host defenses against infection. Albumin forms complexes with bacterial toxins, limiting their pathogenic effects^28,29^. Secondly, albumin stimulates pulmonary surfactant production by type II alveolar cells. Surfactants lower alveolar surface tension and prevent atelectasis. Diminished albumin synthesis leads to reduced surfactant levels, resulting in poor lung compliance^30,31^. Thirdly, low albumin levels lower plasma oncotic pressure and compound surgically-induced increases in capillary permeability, permitting fluid extravasation into the pulmonary interstitium and alveoli, precipitating pulmonary edema^32,33^. Additionally, the ensuing edema and exudation can precipitate respiratory failure and ventilation/perfusion mismatch, engendering hypoxemia—an environment in which bacteria proliferate readily^34,35^. Finally, postoperative malnutrition exacerbates albumin depletion and impedes wound healing and recovery, thereby amplifying infection susceptibility^36^.

Several prior studies have also identified associations between hyperglycemia and increased risk of POP across surgical settings. A large study by Ana López-de-Andrés et al. included 117,665 hospitalized patients and found a significant increase in the probability of POP in patients with hyperglycemia (OR 1.21, 95% CI 1.03–1.42)^37^. Anna Frisch et al. found that in both diabetes patients and non-diabetes patients, perioperative hyperglycemia in non-cardiac surgery was associated with the risk of POP^38^. Tang et al. found even after multiple regression adjustments and PSM adjustment, individuals with hyperglycemia exhibited a substantially higher risk of developing POP in comparison to those with normoglycemia (OR 2.090, 95% CI 1.135–3.846, *p* = 0.016)^39^. Yauhen et al. found that in patients undergoing elective abdominal aortic aneurysm repair, the presence of postoperative hyperglycemia was associated with adverse events, including an increased risk of POP (95% CI 1.68–2.98)^40^.

Firstly, hyperglycemia hinders the functionality of neutrophils, impairing their ability to migrate, phagocytose, and intracellularly eliminate pathogens, rendering diabetic individuals more susceptible to bacterial and fungal pneumonias^41,42^. Secondly, elevated blood glucose levels diminish T-lymphocyte responses, leading to reduced activation and proliferation of lymphocytes crucial for effective cell-mediated immunity against viral and fungal pneumonias^43,44^. Moreover, hyperglycemia promotes increased adherence of pathogens, such as staphylococcus aureus, to respiratory epithelial cells, facilitating bacterial colonization and infection^45,46^. Additionally, neuropathy induced by hyperglycemia can affect the vagus nerve, resulting in a weakened cough reflex, thereby reducing the expulsion of pathogens from the respiratory tract^47^. Lastly, chronic hyperglycemia induces the accumulation of advanced glycation end-products (AGEs), which, upon binding to receptors, impede tissue healing and repair mechanisms in the lungs, ultimately delaying the resolution of pneumonia^48^. These interconnected mechanisms underscore the heightened vulnerability of hyperglycemic patients to various types of pneumonia.

Interestingly, in the interaction analysis, we observed a certain association between GAR and hypertension. However, according to relevant literature reports, false positives may occur when analyzing multiple subgroups^49^. Therefore, we need to further investigate the observed interaction between GAR and hypertension to confirm its authenticity. This will help us gain a more comprehensive understanding of the nature of this association.

Given the above previous findings including clinical research and research on pathophysiological mechanisms, both ALB and hyperglycemia are associated with the occurrence of POP, especially in elderly patients. Our results are consistent with the above research. We explored for the first time the relationship between GAR and POP in elderly patients with hip fractures. Compared to using glucose or albumin levels alone, the GAR integrates information from both biomarkers into a unified measure that may have greater utility for predicting POP, as evidenced by the significant associations and high discriminatory accuracy observed in this study. The ratio provides a consistent, reproducible preoperative risk indicator that builds upon prior research linking glucose dysregulation and hypoalbuminemia with inflated postoperative infection susceptibility. The introduction of GAR opens the door for future studies of other biomarker combinations while potentially guiding targeted preoperative optimization measures to improve surgical outcomes in this vulnerable population.

However, several limitations in this study need further improvement and exploration. Firstly, our study benefited from a large sample size and adjustment for multiple confounders. Limitations include the single-center retrospective design and lack of external validation. Secondly, while we utilized standard diagnostic criteria, some degree of misclassification is possible given the lack of definitive tests for confirming POP. Third, we did not dynamically re-measure biomarker levels during the course of hospitalization and recovery. Changes in biomarkers over time may also be indicative of POP risk. Moreover, due to the retrospective design, we were unable to comprehensively account for potential confounding factors such as cognitive impairment, as observed in previous studies, which could influence the associations with POP risk.

Recording the timing of POP occurrence may shed light on whether pneumonia is associated with prolonged ICU stays. Additionally, future studies should validate the generalizability and clinical utility of GAR in diverse surgical populations and clinical settings. Multicenter studies across different regions, demographics, comorbidities, and surgical procedures are needed. These validation efforts will help determine if GAR should be adopted as a standard preoperative assessment tool to identify high-risk patients needing optimization.

## Conclusions

Our study is the first time to propose GAR as a new clinical biomarker for predicting POP in elderly patients with hip fractures. This new biomarker is useful for early evaluation of POP. Future clinical studies are needed to validate the utility of this new clinical biomarker for predicting POP.

## Supplementary Information

Supplementary Information.

## Data Availability

All the data used and analyzed during the current study are available from the corresponding author upon reasonable request.

## References

[1] Amarilla-Donoso, F. J. *et al.* Quality of life in elderly people after a hip fracture: a prospective study. *Health Qual. Life Outcomes*10.1186/s12955-020-01314-2 (2020). 32171319 10.1186/s12955-020-01314-2 PMC7071575

[2] Veronese, N. & Maggi, S. Epidemiology and social costs of hip fracture. *Injury* **49**, 1458–1460. 10.1016/j.injury.2018.04.015 (2018). 29699731 10.1016/j.injury.2018.04.015

[3] Schnell, S., Friedman, S. M., Mendelson, D. A., Bingham, K. W. & Kates, S. L. The 1-Year mortality of patients treated in a hip fracture program for elders. *Geriatr. Orthop. Surg. Rehabil.* **1**, 6–14. 10.1177/2151458510378105 (2010). 23569656 10.1177/2151458510378105 PMC3597289

[4] Schneider, A. M., Mucharraz, C., Denyer, S. & Brown, N. M. Prolonged hospital stay after arthroplasty for geriatric femoral neck fractures is associated with increased early mortality risk after discharge. *J . Clin. Orthop. Trauma* **26**, 101785. 10.1016/j.jcot.2022.101785 (2022). 35211374 10.1016/j.jcot.2022.101785 PMC8844821

[5] Garcia, A. E. *et al.* Patient variables which may predict length of stay and hospital costs in elderly patients with hip fracture. *J. Orthop. Trauma* **26**, 620–623. 10.1097/BOT.0b013e3182695416 (2012). 22832431 10.1097/BOT.0b013e3182695416

[6] Åhman, R. *et al.* Determinants of mortality after hip fracture surgery in Sweden: A registry-based retrospective cohort study. *Sci. Rep.*10.1038/s41598-018-33940-8 (2018). 30356058 10.1038/s41598-018-33940-8 PMC6200788

[7] Tian, Y. *et al.* Incidence and risk factors for postoperative pneumonia following surgically treated hip fracture in geriatric patients: A retrospective cohort study. *J. Orthop. Surg. Res.*10.1186/s13018-022-03071-y (2022). 35331285 10.1186/s13018-022-03071-y PMC8944015

[8] Han, S.-B., Kim, S.-B. & Shin, K.-H. Risk factors for postoperative pneumonia in patients undergoing hip fracture surgery: A systematic review and meta-analysis. *BMC Musculoskel. Disord.*10.1186/s12891-022-05497-1 (2022). 10.1186/s12891-022-05497-1 PMC9174025 35676675

[9] Gao, Y. C. *et al.* What are risk factors of postoperative pneumonia in geriatric individuals after hip fracture surgery: A systematic review and meta-analysis. *Orthop. Surg.* **15**, 38–52. 10.1111/os.13631 (2022). 36519396 10.1111/os.13631 PMC9837248

[10] Schneider, A. M., Denyer, S. & Brown, N. M. Risk factors associated with extended length of hospital stay after geriatric hip fracture. *JAAOS Glob. Res. Rev.* **5**, e2100073. 10.5435/JAAOSGlobal-D-21-00073 (2021). 10.5435/JAAOSGlobal-D-21-00073 PMC8099404 33945514

[11] Salarbaks, A. M., Lindeboom, R. & Nijmeijer, W. Pneumonia in hospitalized elderly hip fracture patients: the effects on length of hospital-stay, in-hospital and thirty-day mortality and a search for potential predictors. *Injury* **51**, 1846–1850. 10.1016/j.injury.2020.05.017 (2020). 32482422 10.1016/j.injury.2020.05.017

[12] Jang, S.-Y. *et al.* Effect of pneumonia on all-cause mortality after elderly hip fracture: A Korean nationwide cohort study. *J. Korean Med. Sci.*10.3346/jkms.2020.35.e9 (2020). 31920015 10.3346/jkms.2020.35.e9 PMC6955432

[13] Schwartz, J. *et al.* Pre-operative patient optimization to prevent postoperative pulmonary complications-Insights and roles for the respiratory therapist: A narrative review. *Can. J. Respir. Ther.* **56**, 79–85. 10.29390/cjrt-2020-029 (2020). 33304993 10.29390/cjrt-2020-029 PMC7717076

[14] Society, A. T. & America, I. D. Guidelines for the management of adults with hospital-acquired, ventilator-associated, and healthcare-associated pneumonia. *Am. J. Resp. Crit. Care Med.* **171**, 388–416. 10.1164/rccm.200405-644ST (2005). 15699079 10.1164/rccm.200405-644ST

[15] Menzel, A. *et al.* Common and novel markers for measuring inflammation and oxidative stress ex vivo in research and clinical practice—Which to use regarding disease outcomes?. *Antioxidants* **10**, 414. 10.3390/antiox10030414 (2021). 33803155 10.3390/antiox10030414 PMC8001241

[16] Abu Elyazed, M. M. & El Sayed Zaki, M. Value of procalcitonin as a biomarker for postoperative hospital-acquired pneumonia after abdominal surgery. *Korean J. Anesthesiol.* **70**, 177. 10.4097/kjae.2017.70.2.177 (2017). 28367288 10.4097/kjae.2017.70.2.177 PMC5370307

[17] Yao, W., Wang, W., Tang, W., Lv, Q. & Ding, W. Neutrophil-to-lymphocyte ratio (NLR), platelet-to-lymphocyte ratio (PLR), and systemic immune inflammation index (SII) to predict postoperative pneumonia in elderly hip fracture patients. *J . Orthop. Surg. Res.*10.1186/s13018-023-04157-x (2023). 37697317 10.1186/s13018-023-04157-x PMC10496383

[18] Menzel, A. *et al.* Common and novel markers for measuring inflammation and oxidative stress ex vivo in research and clinical practice-which to use regarding disease outcomes?. *Antioxidants* **10**, 414. 10.3390/antiox10030414 (2021). 33803155 10.3390/antiox10030414 PMC8001241

[19] He, J. *et al.* Glucose-albumin ratio as new biomarker for predicting mortality after intracerebral hemorrhage. *Neurosurg. Rev.*10.1007/s10143-023-02002-7 (2023). 37074539 10.1007/s10143-023-02002-7

[20] Berbudi, A., Rahmadika, N., Tjahjadi, A. I. & Ruslami, R. Type 2 diabetes and its impact on the immune system. *Curr. Diabetes Rev.* **16**, 442–449. 10.2174/1573399815666191024085838 (2020). 31657690 10.2174/1573399815666191024085838 PMC7475801

[21] Ulldemolins, M., Roberts, J. A., Rello, J., Paterson, D. L. & Lipman, J. The effects of hypoalbuminaemia on optimizing antibacterial dosing in critically Ill patients. *Clin. Pharmacokinetics* **50**, 99–110. 10.2165/11539220-000000000-00000 (2011). 10.2165/11539220-000000000-00000 21142293

[22] Peduzzi, P., Concato, J., Feinstein, A. R. & Holford, T. R. Importance of events per independent variable in proportional hazards regression analysis: II: Accuracy and precision of regression estimates. *J. Clin. Epidemiol.* **48**, 1503–1510. 10.1016/0895-4356(95)00048-8 (1995). 8543964 10.1016/0895-4356(95)00048-8

[23] Concato, J., Peduzzi, P., Holford, T. R. & Feinstein, A. R. Importance of events per independent variable in proportional hazards analysis: I: Background, goals, and general strategy. *J. Clin. Epidemiol.* **48**, 1495–1501. 10.1016/0895-4356(95)00510-2 (1995). 8543963 10.1016/0895-4356(95)00510-2

[24] Harrington, D. *et al.* New guidelines for statistical reporting in the journal. *New Engl. J. Med.* **381**, 285–286. 10.1056/NEJMe1906559 (2019). 31314974 10.1056/NEJMe1906559

[25] Wang, Y. *et al.* <p>Preoperative serum albumin level as a predictor of postoperative pneumonia after femoral neck fracture surgery in a geriatric population</p&gt. *Clin. Interv. Aging* **14**, 2007–2016. 10.2147/cia.S231736 (2019). 32009780 10.2147/CIA.S231736 PMC6859085

[26] Tian, Y. *et al.* Relationship between preoperative hypoalbuminemia and postoperative pneumonia following geriatric hip fracture surgery: A propensity-score matched and conditional logistic regression analysis. *Clin. Interv. Aging* **17**, 495–503. 10.2147/cia.S352736 (2022). 35444412 10.2147/CIA.S352736 PMC9013674

[27] Lee, S. H. & Kim, K. U. Risk factors for postoperative pneumonia in the elderly following hip fracture surgery: A systematic review and meta-analysis. *Geriatr. Orthop. Surg. Rehabil.* **13**, 215145932210838. 10.1177/21514593221083825 (2022). 10.1177/21514593221083825 PMC9133882 35634259

[28] Soeters, P. B., Wolfe, R. R. & Shenkin, A. Hypoalbuminemia: Pathogenesis and clinical significance. *J. Parent. Enteral Nutr.* **43**, 181–193. 10.1002/jpen.1451 (2019). 10.1002/jpen.1451 PMC7379941 30288759

[29] Wilde, B. & Katsounas, A. Immune dysfunction and albumin-related immunity in liver cirrhosis. *Mediat. Inflamm.* **2019**, 7537649. 10.1155/2019/7537649 (2019). 10.1155/2019/7537649 PMC6410448 30930689

[30] Nkadi, P. O., Merritt, T. A. & Pillers, D.-A.M. An overview of pulmonary surfactant in the neonate: Genetics, metabolism, and the role of surfactant in health and disease. *Mol. Genet. Metab.* **97**, 95–101. 10.1016/j.ymgme.2009.01.015 (2009). 19299177 10.1016/j.ymgme.2009.01.015 PMC2880575

[31] Wang, S. *et al.* The role of pulmonary surfactants in the treatment of acute respiratory distress syndrome in COVID-19. *Front. Pharmacol.*10.3389/fphar.2021.698905 (2021). 34267664 10.3389/fphar.2021.698905 PMC8276044

[32] McNeil, J. B. *et al.* Linear association between hypoalbuminemia and increased risk of acute respiratory distress syndrome in critically Ill adults. *Crit. Care Explor.* **3**, e0527. 10.1097/cce.0000000000000527 (2021). 34549190 10.1097/CCE.0000000000000527 PMC8443821

[33] Thirdly, low albumin levels lower plasma oncotic pressure and compound surgically-induced increases in capillary permeability, permitting fluid extravasation into the pulmonary interstitium and alveoli, precipit.pdf.

[34] Jeong, J. S., Jun, J. H., Song, H. J. & Choi, S. H. Acute pulmonary edema due to hypoxia during a difficult intubation in a rheumatoid arthritis patient. *Korean J. Anesthesiol.* **67**, S74. 10.4097/kjae.2014.67.S.S74 (2014). 25598917 10.4097/kjae.2014.67.S.S74 PMC4295991

[35] Khademi, S. *et al.* Hypoxia mediated pulmonary edema: Potential influence of oxidative stress, sympathetic activation and cerebral blood flow. *BMC Physiol.* **15**, 4. 10.1186/s12899-015-0018-4 (2015). 26449218 10.1186/s12899-015-0018-4 PMC4599206

[36] Stechmiller, J. K. Understanding the role of nutrition and wound healing. *Nutr. Clin. Pract.* **25**, 61–68. 10.1177/0884533609358997 (2010). 20130158 10.1177/0884533609358997

[37] López-de-Andrés, A. *et al.* Type 2 diabetes and postoperative pneumonia: An observational, population-based study using the Spanish Hospital Discharge Database, 2001–2015. *PLOS ONE*10.1371/journal.pone.0211230 (2019). 30726277 10.1371/journal.pone.0211230 PMC6364970

[38] Frisch, A. *et al.* Prevalence and clinical outcome of hyperglycemia in the perioperative period in noncardiac surgery. *Diabetes Care* **33**, 1783–1788. 10.2337/dc10-0304 (2010). 20435798 10.2337/dc10-0304 PMC2909062

[39] Tang, W., Yao, W., Wang, W., Lv, Q. & Ding, W. Association between admission hyperglycemia and postoperative pneumonia in geriatric patients with hip fractures. *BMC Musculoskel. Disorders*10.1186/s12891-023-06829-5 (2023). 10.1186/s12891-023-06829-5 PMC10472715 37658378

[40] Tarbunou, Y. A., Smith, J. B., Kruse, R. L. & Vogel, T. R. Outcomes associated with hyperglycemia after abdominal aortic aneurysm repair. *J. Vasc. Surg.* **69**, 763-773.e763. 10.1016/j.jvs.2018.05.240 (2019). 30154015 10.1016/j.jvs.2018.05.240 PMC6389385

[41] Thimmappa, P. Y., Vasishta, S., Ganesh, K., Nair, A. S. & Joshi, M. B. Neutrophil (dys)function due to altered immuno-metabolic axis in type 2 diabetes: Implications in combating infections. *Human Cell* **36**, 1265–1282. 10.1007/s13577-023-00905-7 (2023). 37115481 10.1007/s13577-023-00905-7 PMC10284735

[42] Dowey, R., Iqbal, A., Heller, S. R., Sabroe, I. & Prince, L. R. A bittersweet response to infection in diabetes; targeting neutrophils to modify inflammation and improve host immunity. *Front. Immunol.*10.3389/fimmu.2021.678771 (2021). 34149714 10.3389/fimmu.2021.678771 PMC8209466

[43] Jacobs, S. R. *et al.* Glucose uptake is limiting in T cell activation and requires CD28-Mediated Akt-dependent and independent pathways. *J. Immunol.* **180**, 4476–4486. 10.4049/jimmunol.180.7.4476 (2008). 18354169 10.4049/jimmunol.180.7.4476 PMC2593791

[44] MacIver, N. J. *et al.* Glucose metabolism in lymphocytes is a regulated process with significant effects on immune cell function and survival. *J. Leukocyte Biol.* **84**, 949–957. 10.1189/jlb.0108024 (2008). 18577716 10.1189/jlb.0108024 PMC2638731

[45] Thurlow, L. R., Stephens, A. C., Hurley, K. E. & Richardson, A. R. Lack of nutritional immunity in diabetic skin infections promotes *Staphylococcus aureus* virulence. *Sci. Adv.*10.1126/sciadv.abc5569 (2020). 33188027 10.1126/sciadv.abc5569 PMC7673755

[46] Garnett, J. P. *et al.* Metformin reduces airway glucose permeability and hyperglycaemia-inducedStaphylococcus aureusload independently of effects on blood glucose. *Thorax* **68**, 835–845. 10.1136/thoraxjnl-2012-203178 (2013). 23709760 10.1136/thoraxjnl-2012-203178 PMC3756442

[47] Al-Biltagi, M., Bediwy, A. S. & Saeed, N. K. Cough as a neurological sign: What a clinician should know. *World J. Crit. Care Med.* **11**, 115–128. 10.5492/wjccm.v11.i3.115 (2022). 36331984 10.5492/wjccm.v11.i3.115 PMC9136724

[48] Jensen, A. V. *et al.* The impact of blood glucose on community-acquired pneumonia: A retrospective cohort study. *ERJ Open Res.* **3**, 00114–02016. 10.1183/23120541.00114-2016 (2017). 28656133 10.1183/23120541.00114-2016 PMC5478863

[49] Harrington, D. *et al.* New guidelines for statistical reporting in the journal. *New England J. Med.* **381**, 285–286. 10.1056/NEJMe1906559 (2019). 31314974 10.1056/NEJMe1906559

