# Supplementary material for: Glucose-albumin ratio (GAR) as a novel biomarker for predicting postoperative pneumonia (POP) in older adults with hip fractures
Source: Sci Rep. 2024 Nov 4;14:26637. doi: 10.1038/s41598-024-60390-2 (PMC11535218; doi:10.1038/s41598-024-60390-2)
Supplement: Supplementary file 1 — Supplementary Information. [file 41598_2024_60390_MOESM1_ESM.docx]

**Appendix:**

**eFigure 1 Flow diagram of patients included in the study**

**eFigure 2 The cutoff values of individual and combined blood-based biomarkers for predicting POP were examined by ROC curve analysis**

**eFigure 3 Subgroup analysis of adjustment association between GAR and POP after propensity score matching.**

**eTable 1 Univariate and multivariate regression analyses of risk factors for POP (WBC -POP)**

**eTable 2 Univariate and multivariate regression analyses of risk factors for POP (NEU-POP)**

**eTable 3 Univariate and multivariate regression analyses of risk factors for POP (Glucose-POP)**

**eTable 4 Univariate and multivariate regression analyses of risk factors for POP (ALB-POP)**

**eTable 5 Comparison of the incidence of POP before and after PSM based on GAR.**

**eTable 6 Patient characteristics before and after propensity score matching by GAR cutoffs**

**eTable 7 Patient characteristics before and after propensity score matching by GAR(Q1 [< 0.137] vs. Q2[0.137-0.164])**

**eTable8 Patient characteristics before and after propensity score matching by GAR(Q1 [< 0.137] vs. Q3[0.164-0.206])**

**eTable9 Patient characteristics before and after propensity score matching by GAR(Q1 [ < 0.137] vs. Q4[ ≥ 0.206])**

**
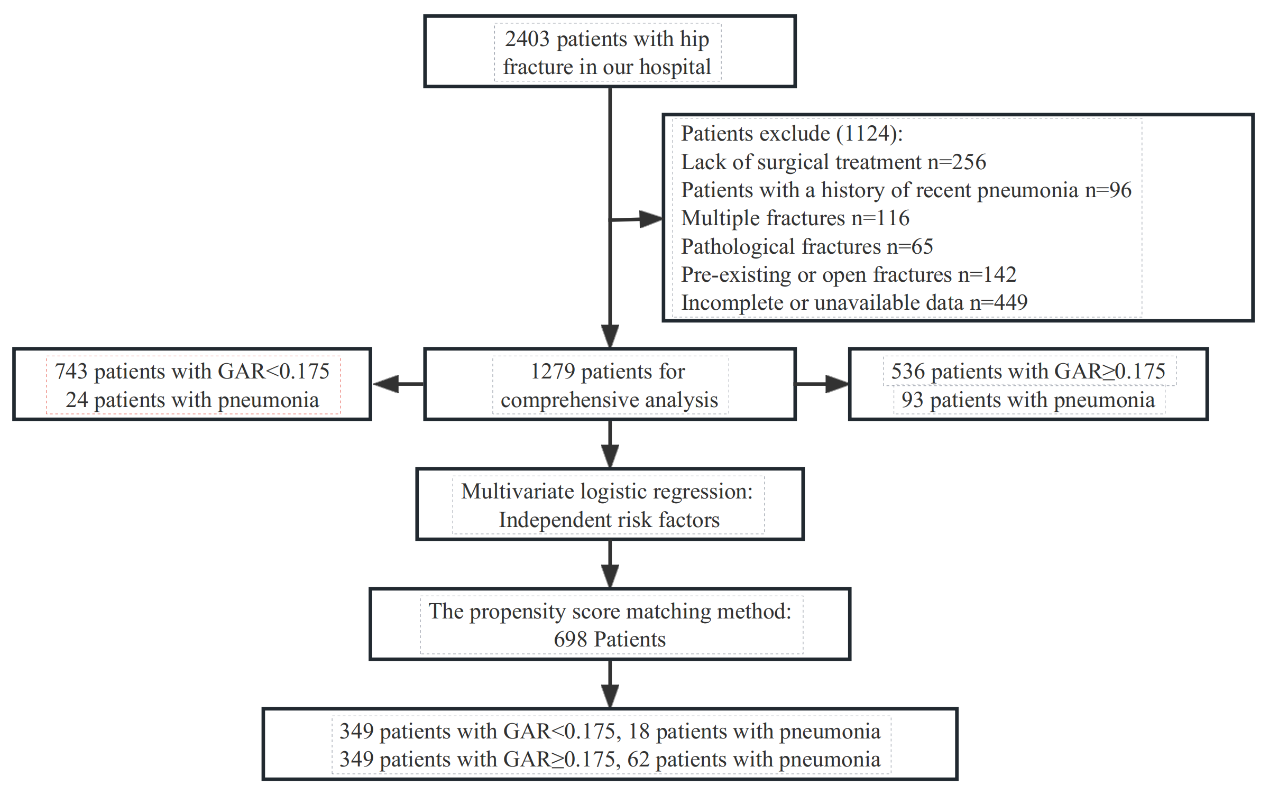
**

**eFigure1 Flow diagram of patients included in the study.**


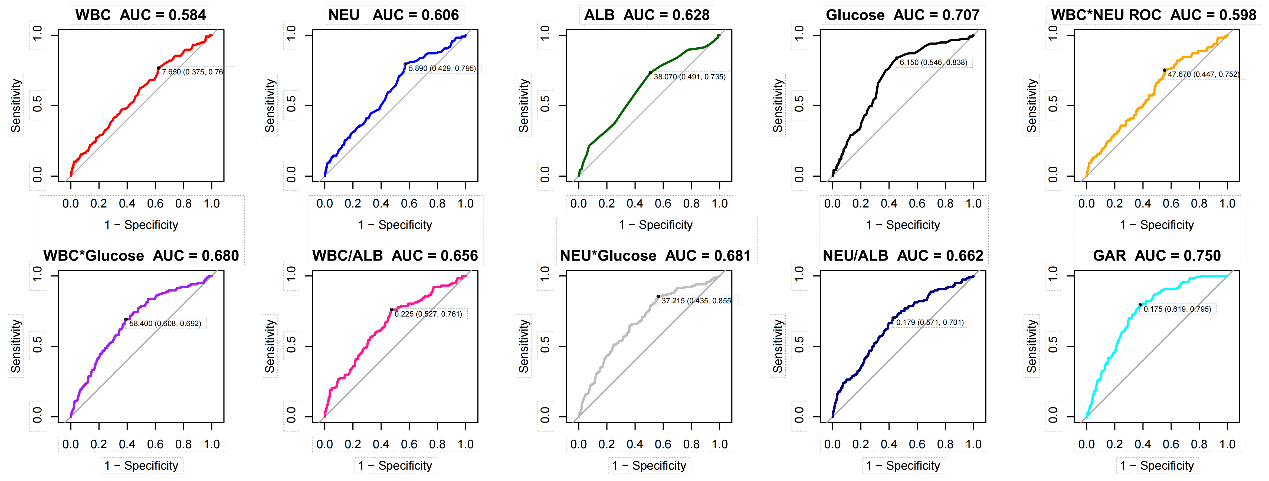


**eFigure2 Subgroup analysis of adjustment association between glucose and POP a after propensity score matching**

**
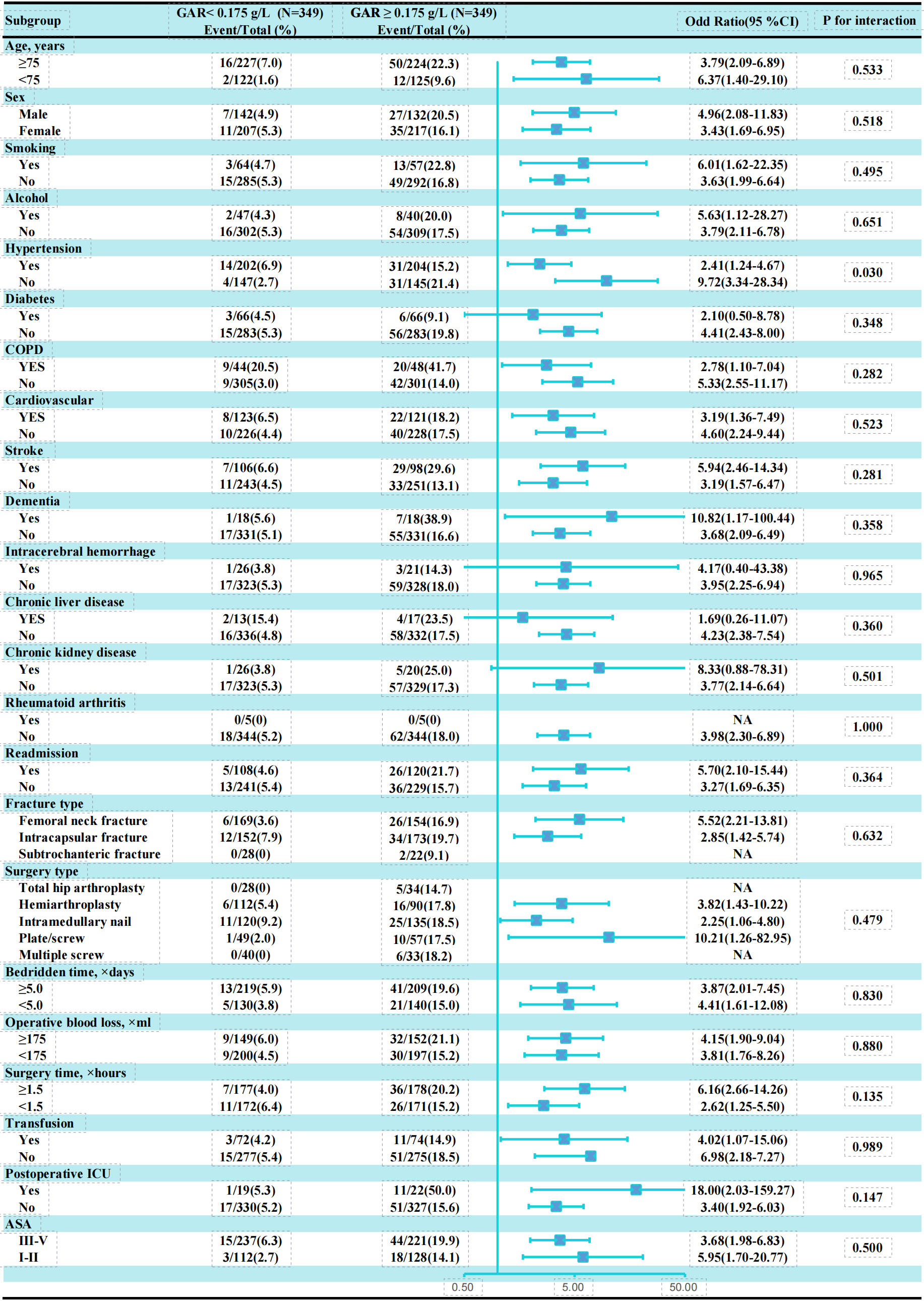
**

**eFigure 3 Subgroup analysis of adjustment association between GAR and POP after propensity score matching.**

**.**

**eTable1 Univariate and multivariate regression analyses of risk factors for POP (WBC -POP)**

| Characteristics | Univariate | | | Multivariate | | |
| --- | --- | --- | --- | --- | --- | --- |
|  | OR | 95%CI | P-value | OR | 95%CI | P-value |
| Age | 1.08 | 1.05-1.10 | <0.001 | 1.06 | 1.03-1.08 | 0.003 |
| Female gender | 0.93 | 0.67-1.45 | 0.926 | <NA> | <NA> | <NA> |
| Smoking | 1.07 | 0.65-1.76 | 0.785 | <NA> | <NA> | <NA> |
| Alcohol | 1.04 | 0.58-1.88 | 0.889 | <NA> | <NA> | <NA> |
| Hypertension | 1.43 | 0.97-2.10 | 0.070 | <NA> | <NA> | <NA> |
| Diabetes | 1.40 | 0.92-2.14 | 0.118 | <NA> | <NA> | <NA> |
| COPD | 7.23 | 4.75-11.01 | <0.001 | 5.43 | 3.41-8.64 | <0.001 |
| Cardiovascular | 1.85 | 1.25-2.72 | 0.002 | 0.90 | 0.56-1.41 | 0.644 |
| Stroke | 2.34 | 1.58-3.44 | <0.001 | 1.82 | 1.17-2.84 | 0.008 |
| Dementia | 2.40 | 1.13-5.09 | 0.022 | 1.56 | 0.67-3.63 | 0.304 |
| Intracerebral hemorrhage | 1.11 | 0.50-2.48 | 0.799 | <NA> | <NA> | <NA> |
| Chronic liver disease | 2.17 | 1.07-4.41 | 0.032 | 1.66 | 0.72-3.86 | 0.236 |
| Chronic kidney disease | 2.39 | 1.24-4.62 | 0.009 | 1.64 | 0.76-3.56 | 0.211 |
| Rheumatoid arthritis | NA | NA | NA | <NA> | <NA> | <NA> |
| Readmission | 1.87 | 1.27-2.76 | 0.002 | 1.40 | 0.90-2.20 | 0.139 |
| Fracture type | 0.71 | 0.53-0.96 | 0.027 | 0.86 | 0.60-1.23 | 0.86 |
| Surgery type | 0.92 | 0.79-1.07 | 0.285 | <NA> | <NA> | <NA> |
| Bedridden time | 1.10 | 1.06-1.14 | <0.001 | 1.07 | 1.03-1.11 | 0.002 |
| Operative blood loss | 1.00 | 1.00-1.00 | 0.410 | <NA> | <NA> | <NA> |
| Surgery time | 1.08 | 0.87-1.35 | 0.493 | <NA> | <NA> | <NA> |
| Transfusion | 1.13 | 0.69-1.85 | 0.640 | <NA> | <NA> | <NA> |
| Postoperative ICU | 3.86 | 2.14-6.96 | <0.001 | 2.77 | 1.42-5.41 | 0.003 |
| ASA | 2.89 | 1.85-4.52 | <0.001 | 1.30 | 0.78-2.16 | 0.309 |
| WBC | 1.12 | 1.05-1.18 | <0.001 | 1.09 | 1.02-1.17 | <0.010 |

*p*-value: *p* < 0.05 indicates that the risk factor is statistically significantly associated with the POP outcome

Abbreviations: ASA, American Society of Anesthesiologists score; WBC, White Blood Cell

**eTable2 Univariate and multivariate regression analyses of risk factors for POP (NEU-POP)**

| Characteristics | Univariate | | | Multivariate | | |
| --- | --- | --- | --- | --- | --- | --- |
|  | OR | 95%CI | P-value | OR | 95%CI | P-value |
| Age | 1.08 | 1.05-1.10 | <0.001 | 1.06 | 1.03-1.09 | <0.001 |
| Female gender | 0.93 | 0.67-1.45 | 0.926 | <NA> | <NA> | <NA> |
| Smoking | 1.07 | 0.65-1.76 | 0.785 | <NA> | <NA> | <NA> |
| Alcohol | 1.04 | 0.58-1.88 | 0.889 | <NA> | <NA> | <NA> |
| Hypertension | 1.43 | 0.97-2.10 | 0.070 | <NA> | <NA> | <NA> |
| Diabetes | 1.40 | 0.92-2.14 | 0.118 | <NA> | <NA> | <NA> |
| COPD | 7.23 | 4.75-11.01 | <0.001 | 5.34 | 3.35-8.50 | <0.001 |
| Cardiovascular | 1.85 | 1.25-2.72 | 0.002 | 0.91 | 0.58-1.42 | 0.666 |
| Stroke | 2.34 | 1.58-3.44 | <0.001 | 1.82 | 1.16-2.83 | 0.009 |
| Dementia | 2.40 | 1.13-5.09 | 0.022 | 1.54 | 0.66-3.58 | 0.319 |
| Intracerebral hemorrhage | 1.11 | 0.50-2.48 | 0.799 | <NA> | <NA> | <NA> |
| Chronic liver disease | 2.17 | 1.07-4.41 | 0.032 | 1.66 | 0.71-3.86 | 0.242 |
| Chronic kidney disease | 2.39 | 1.24-4.62 | 0.009 | 1.63 | 0.75-3.54 | 0.219 |
| Rheumatoid arthritis | NA | NA | NA | <NA> | <NA> | <NA> |
| Readmission | 1.87 | 1.27-2.76 | 0.002 | 1.41 | 0.90-2.21 | 0.135 |
| Fracture type | 0.71 | 0.53-0.96 | 0.027 | 0.97 | 0.67-1.40 | 0.87 |
| Surgery type | 0.92 | 0.79-1.07 | 0.285 | <NA> | <NA> | <NA> |
| Bedridden time | 1.10 | 1.06-1.14 | <0.001 | 1.07 | 1.03-1.11 | 0.002 |
| Operative blood loss | 1.00 | 1.00-1.00 | 0.410 | <NA> | <NA> | <NA> |
| Surgery time | 1.08 | 0.87-1.35 | 0.493 | <NA> | <NA> | <NA> |
| Transfusion | 1.13 | 0.69-1.85 | 0.640 | <NA> | <NA> | <NA> |
| Postoperative ICU | 3.86 | 2.14-6.96 | <0.001 | 2.77 | 1.42-5.40 | 0.003 |
| ASA | 2.89 | 1.85-4.52 | <0.001 | 1.29 | 0.78-2.14 | 0.323 |
| NEU | 1.14 | 1.07-1.21 | <0.001 | 1.11 | 1.03-1.18 | 0.004 |

*p*-value: *p* < 0.05 indicates that the risk factor is statistically significantly associated with the POP outcome

Abbreviations: ASA, American Society of Anesthesiologists score; NEU, neutrophil

**eTable3 Univariate and multivariate regression analyses of risk factors for POP (Glucose-POP)**

| Characteristics | Univariate | | | Multivariate | | |
| --- | --- | --- | --- | --- | --- | --- |
|  | OR | 95%CI | P-value | OR | 95%CI | P-value |
| Age | 1.08 | 1.05-1.10 | <0.001 | 1.05 | 1.03-1.08 | <0.001 |
| Female gender | 0.93 | 0.67-1.45 | 0.926 | <NA> | <NA> | <NA> |
| Smoking | 1.07 | 0.65-1.76 | 0.785 | <NA> | <NA> | <NA> |
| Alcohol | 1.04 | 0.58-1.88 | 0.889 | <NA> | <NA> | <NA> |
| Hypertension | 1.43 | 0.97-2.10 | 0.070 | <NA> | <NA> | <NA> |
| Diabetes | 1.40 | 0.92-2.14 | 0.118 | <NA> | <NA> | <NA> |
| COPD | 7.23 | 4.75-11.01 | <0.001 | 5.71 | 3.58-9.12 | <0.001 |
| Cardiovascular | 1.85 | 1.25-2.72 | 0.002 | 0.84 | 0.54-1.32 | 0.454 |
| Stroke | 2.34 | 1.58-3.44 | <0.001 | 1.74 | 1.12-2.72 | 0.014 |
| Dementia | 2.40 | 1.13-5.09 | 0.022 | 1.68 | 0.72-3.96 | 0.233 |
| Intracerebral hemorrhage | 1.11 | 0.50-2.48 | 0.799 | <NA> | <NA> | <NA> |
| Chronic liver disease | 2.17 | 1.07-4.41 | 0.032 | 1.73 | 0.76-3.95 | 0.195 |
| Chronic kidney disease | 2.39 | 1.24-4.62 | 0.009 | 1.66 | 0.77-3.60 | 0.197 |
| Rheumatoid arthritis | NA | NA | NA | <NA> | <NA> | <NA> |
| Readmission | 1.87 | 1.27-2.76 | 0.002 | 1.35 | 0.86-2.11 | 0.194 |
| Fracture type | 0.71 | 0.53-0.96 | 0.027 | 0.85 | 0.59-1.21 | 0.356 |
| Surgery type | 0.92 | 0.79-1.07 | 0.285 | <NA> | <NA> | <NA> |
| Bedridden time | 1.10 | 1.06-1.14 | <0.001 | 1.06 | 1.02-1.11 | 0.005 |
| Operative blood loss | 1.00 | 1.00-1.00 | 0.410 | <NA> | <NA> | <NA> |
| Surgery time | 1.08 | 0.87-1.35 | 0.493 | <NA> | <NA> | <NA> |
| Transfusion | 1.13 | 0.69-1.85 | 0.640 | <NA> | <NA> | <NA> |
| Postoperative ICU | 3.86 | 2.14-6.96 | <0.001 | 3.01 | 1.54-5.86 | 0.001 |
| ASA | 2.89 | 1.85-4.52 | <0.001 | 1.231 | 0.79-2.17 | 0.30 |
| Glucose | 1.16 | 1.10-1.22 | <0.001 | 1.13 | 1.06-1.20 | <0.001 |

*p*-value: *p* < 0.05 indicates that the risk factor is statistically significantly associated with the POP outcome

Abbreviations: ASA, American Society of Anesthesiologists score

**eTable4 Univariate and multivariate regression analyses of risk factors for POP (ALB-POP)**

| Characteristics | Univariate | | | Multivariate | | |
| --- | --- | --- | --- | --- | --- | --- |
|  | OR | 95%CI | P-value | OR | 95%CI | P-value |
| Age | 1.08 | 1.05-1.10 | <0.001 | 1.04 | 1.01-1.07 | 0.004 |
| Female gender | 0.93 | 0.67-1.45 | 0.926 | <NA> | <NA> | <NA> |
| Smoking | 1.07 | 0.65-1.76 | 0.785 | <NA> | <NA> | <NA> |
| Alcohol | 1.04 | 0.58-1.88 | 0.889 | <NA> | <NA> | <NA> |
| Hypertension | 1.43 | 0.97-2.10 | 0.070 | <NA> | <NA> | <NA> |
| Diabetes | 1.40 | 0.92-2.14 | 0.118 | <NA> | <NA> | <NA> |
| COPD | 7.23 | 4.75-11.01 | <0.001 | 5.49 | 3.45-8.72 | <0.001 |
| Cardiovascular | 1.85 | 1.25-2.72 | 0.002 | 0.92 | 0.59-1.44 | 0.922 |
| Stroke | 2.34 | 1.58-3.44 | <0.001 | 1.69 | 1.08-2.64 | 0.021 |
| Dementia | 2.40 | 1.13-5.09 | 0.022 | 1.42 | 0.60-3.34 | 0.428 |
| Intracerebral hemorrhage | 1.11 | 0.50-2.48 | 0.799 | <NA> | <NA> | <NA> |
| Chronic liver disease | 2.17 | 1.07-4.41 | 0.032 | 1.57 | 0.69-3.59 | 0.286 |
| Chronic kidney disease | 2.39 | 1.24-4.62 | 0.009 | 1.66 | 0.77-3.56 | 0.193 |
| Rheumatoid arthritis | NA | NA | NA | <NA> | <NA> | <NA> |
| Readmission | 1.87 | 1.27-2.76 | 0.002 | 1.38 | 0.88-2.17 | 0.160 |
| Fracture type | 0.71 | 0.53-0.96 | 0.027 | 0.86 | 0.60-2.17 | 0.418 |
| Surgery type | 0.92 | 0.79-1.07 | 0.285 | <NA> | <NA> | <NA> |
| Bedridden time | 1.10 | 1.06-1.14 | <0.001 | 1.07 | 1.02-1.11 | 0.003 |
| Operative blood loss | 1.00 | 1.00-1.00 | 0.410 | <NA> | <NA> | <NA> |
| Surgery time | 1.08 | 0.87-1.35 | 0.493 | <NA> | <NA> | <NA> |
| Transfusion | 1.13 | 0.69-1.85 | 0.640 | <NA> | <NA> | <NA> |
| Postoperative ICU | 3.86 | 2.14-6.96 | <0.001 | 2.62 | 1.35-5.08 | 0.004 |
| ASA | 2.89 | 1.85-4.52 | <0.001 | 1.36 | 0.82-2.25 | 0.236 |
| ALB | 1.14 | 1.10-1.18 | <0.001 | 1.06 | 1.01-1.11 | 0.010 |

*p*-value: *p* < 0.05 indicates that the risk factor is statistically significantly associated with the POP outcome

Abbreviations: ASA, American Society of Anesthesiologists score; ALB, Albumin

**eTable 5** **Comparison of the incidence of POP before and after PSM based on GAR.**

| Categories | Categories | No. (%)  cutoffs | Before PSM | | p | After PSM | | p |
| --- | --- | --- | --- | --- | --- | --- | --- | --- |
|  |  |  | Non-POP  （n=1162） | POP  （n=117） |  | Non-POP  (n=618) | POP  （n=80） |  |
| GAR | Cutoff | < 0.175 | 719 (96.8) | 24 (3.2) | <0.001 | 331 (94.8) | 18(5.2) | <0.001 |
|  |  | ≥ 0.175 | 443 (82.7) | 93 (17.3) |  | 287(82.2) | 62(17.8) |  |
|  | Quartile | Q1( < 0.137) | 317 (93.7) | 3 (0.9) | <0.001 | 100(97.1) | 3(2.9) | <0.001 |
|  |  | Q2(0.137-0.164) | 304 (95.0) | 16 (5.0) |  | 167(93.8) | 11(6.2) |  |
|  |  | Q3(0.164-0.206) | 284 (88.8) | 36 (11.2) |  | 216(88.9) | 27(11.1) |  |
|  |  | Q4( ≥ 0.206) | 257 (81.6) | 62 (19.4) |  | 135(77.6) | 39(22.4) |  |

Abbreviations: GAR, glucose to albumin ratio; p for trend.

**eTable6 Patient characteristics before and after propensity score matching by GAR cutoffs (< 0.175 vs.** **GAR ≥ 0.175)**

| Characteristics | Before matching | | | After matching | | |
| --- | --- | --- | --- | --- | --- | --- |
|  | GAR < 0.175  (n=743) | GAR ≥ 0.175  (n=536) | SMD | GAR < 0.175 (n=349) | GAR ≥ 0.175  (n=349) | SMD |
| Demographic |  |  |  |  |  |  |
| Age ≥75 (n,%) | 321 (43.2) | 358 (66.8) | 0.488 | 227 (65.0) | 224 (64.2) | 0.018 |
| Female gender (n,%) | 421 (56.7) | 350 (65.3) | 0.178 | 207 (59.3) | 217 (62.2) | 0.059 |
| Smoking (n,%) | 142 (19.1) | 76 (14.2) | 0.133 | 64 (18.3) | 57 (16.3) | 0.053 |
| Alcohol (n,%) | 101 (13.6) | 47 (8.8) | 0.153 | 47 (13.5) | 40 (11.5) | 0.061 |
| Comorbidities |  |  |  |  |  |  |
| Hypertension (n,%) | 306 (41.2) | 335 (62.5) | 0.436 | 202 (57.9) | 204 (58.5) | 0.012 |
| Diabetes (n,%) | 68 (9.2) | 229 (42.7) | 0.828 | 66 (18.9) | 66 (18.9) | <0.001 |
| COPD (n,%) | 65 (8.7) | 85 (15.9) | 0.218 | 44 (12.6) | 48 (13.8) | 0.034 |
| Cardiovascular (n,%) | 186 (25.0) | 208 (38.8) | 0.298 | 123 (35.2) | 121 (34.7) | 0.012 |
| Stroke (n,%) | 158 (21.3) | 174 (32.5) | 0.254 | 106 (30.4) | 98 (28.1) | 0.050 |
| Dementia (n,%) | 27 (3.6) | 21 (3.9) | 0.015 | 18 (5.2) | 18 (5.2) | <0.001 |
| Intracerebral hemorrhage (n,%) | 35 (4.7) | 35 (6.5) | 0.079 | 26 (7.4) | 21 (6.0) | 0.057 |
| Chronic liver disease (n,%) | 23 (3.1) | 35 (6.5) | 0.161 | 13 (3.7) | 17 (4.9) | 0.056 |
| Chronic kidney disease (n,%) | 30 (4.0) | 35 (6.5) | 0.111 | 26 (7.4) | 20 (5.7) | 0.069 |
| Rheumatoid arthritis (n,%) | 13 (1.7) | 9 (1.7) | 0.005 | 5 (1.4) | 5 (1.4) | <0.001 |
| Readmission (n,%) | 186 (25.0) | 186 (34.7) | 0.212 | 108 (30.9) | 120 (34.4) | 0.073 |
| Fracture type |  |  |  |  |  |  |
| Femoral neck fracture (n,%) | 468 (63.0) | 216 (40.3) | 0.415 | 169 (48.4) | 154 (44.1) | 0.042 |
| Intertrochanteric fracture (n,%) | 238 (32.0) | 283 (52.8) |  | 152 (43.6) | 173 (49.6) |  |
| Subtrochanteric fracture (n,%) | 37 (5.0) | 37 (6.9) |  | 28 (8.0) | 22 (6.3) |  |
| Surgery type |  |  |  |  |  |  |
| Total hip arthroplasty (n,%) | 106 (14.3) | 56 (10.5) | 0.102 | 28 (8.0) | 34 (9.7) | 0.010 |
| Hemiarthroplasty (n,%) | 200 (26.9) | 122 (22.8) |  | 112 (32.1) | 90 (25.8) |  |
| Intramedullary nail (n,%) | 188 (25.3) | 228 (42.5) |  | 120 (34.4) | 135 (38.7) |  |
| Plate/screw (n,%) | 81 (10.9) | 89 (16.6) |  | 49 (14.0) | 57 (16.3) |  |
| Multiple screws (n,%) | 168 (22.6) | 41 (7.6) |  | 40 (11.5) | 33 (9.5) |  |
| Bedridden time ≥ 5.0 days (n,%) | 370 (49.8) | 354 (66.0) | 0.333 | 219 (62.8) | 209 (59.9) | 0.059 |
| Operative blood loss ≥ 175 ml (n,%) | 277 (37.3) | 242 (45.1) | 0.160 | 149 (42.7) | 152 (43.6) | 0.017 |
| Surgery time ≥ 1.5hours (n,%) | 350 (47.1) | 300 (56.0) | 0.102 | 177 (50.7) | 178 (51.0) |  |
| Transfusion (n,%) | 88 (11.8) | 122 (22.8) | 0.291 | 72 (20.6) | 74 (21.2) | 0.014 |
| Postoperative ICU (n,%) | 24 (3.2) | 42 (7.8) | 0.202 | 19 (5.4) | 22 (6.3) | 0.037 |
| ASA |  |  |  |  |  |  |
| Ⅲ-Ⅴ(n,%) | 354 (47.6) | 300 (56.0) | 0.394 | 237 (67.9) | 221 (63.3) | 0.096 |
| Ⅰ-Ⅱ(n,%) | 393 (52.9) | 236 (44.0) |  | 112 (32.1) | 128 (36.7) |  |

Abbreviations: SMD: Standardized Mean Difference; ASA, American Society of Anesthesiologists score

**eTable7 Patient characteristics before and after propensity score matching by GAR(Q1 [< 0.137] vs. Q2[0.137-0.164])**

| Characteristics | Before matching | | | After matching | | |
| --- | --- | --- | --- | --- | --- | --- |
|  | Q1( < 0.137)  (n= 320) | Q2(0.137-0.164)  (n= 320) | SMD | Q1( < 0.137)  (n=211) | Q2(0.137-0.164)  (n=211) | SMD |
| Demographic |  |  |  |  |  |  |
| Age ≥75 (n,%) | 86 (26.9) | 181 (43.4) | 0.630 | 84 (39.8) | 84 (39.8) | <0.001 |
| Female gender (n,%) | 180 (56.3) | 177 (55.3) | 0.019 | 115 (54.5) | 92 (43.6) | 0.038 |
| Smoking (n,%) | 67 (20.9) | 63 (19.7) | 0.031 | 50 (23.7) | 50 (23.7) | <0.001 |
| Alcohol (n,%) | 36 (11.3) | 54 (16.9) | 0.162 | 34 (16.1) | 33 (15.6) | 0.013 |
| Comorbidities |  |  |  |  |  |  |
| Hypertension (n,%) | 98 (30.6) | 151 (47.2) | 0.344 | 79 (37.4) | 77 (36.5) | 0.02 |
| Diabetes (n,%) | 19 (5.9) | 30 (9.4) | 0.129 | 17 (8.1) | 15 (7.1) | 0.036 |
| COPD (n,%) | 21 (6.6) | 31 (9.7) | 0.114 | 19 (9.0) | 17 (8.1) | 0.034 |
| Cardiovascular (n,%) | 64 (20.0) | 93 (29.1) | 0.211 | 53 (25.1) | 57 (27.0) | 0.043 |
| Stroke (n,%) | 52 (16.3) | 77 (24.1) | 0.195 | 42 (19.9) | 44 (20.9) | 0.023 |
| Dementia (n,%) | 11 (3.4) | 11 (3.4) | <0.001 | 6 (2.8) | 6 (2.8) | <0.001 |
| Intracerebral hemorrhage (n,%) | 10 (3.1) | 16 (5.0) | 0.095 | 8 (3.8) | 7 (3.3) | 0.026 |
| Chronic liver disease (n,%) | 8 (2.5) | 14 (4.4) | 0.103 | 8 (3.8) | 9 (4.3) | 0.024 |
| Chronic kidney disease (n,%) | 10 (3.1) | 17 (5.3) | 0.109 | 6 (2.8) | 8 (3.8) | 0.053 |
| Rheumatoid arthritis (n,%) | 6 (1.9) | 6 (1.9) | <0.001 | 5 (2.4) | 5 (2.4) | <0.001 |
| Readmission (n,%) | 69 (21.6) | 89 (27.8) | 0.145 | 53 (25.1) | 51 (24.2) | 0.022 |
| Fracture type |  |  |  |  |  |  |
| Femoral neck fracture (n,%) | 228 (71.3) | 190 (59.4) | 0.183 | 140 (66.4) | 140 (66.4) | 0.008 |
| Intertrochanteric fracture (n,%) | 74 (23.1) | 116 (36.3) |  | 60 (28.4) | 59 (28.0) |  |
| Subtrochanteric fracture (n,%) | 18 (5.6) | 14 (4.4) |  | 11 (5.2) | 12 (5.7) |  |
| Surgery type |  |  |  |  |  |  |
| Total hip arthroplasty (n,%) | 62 (19.4) | 32 (10.0) | 0.148 | 41 (19.4) | 24 (11.4) | 0.010 |
| Hemiarthroplasty (n,%) | 64 (20.0) | 105 (32.8) |  | 48 (22.7) | 69 (32.7) |  |
| Intramedullary nail (n,%) | 62 (19.4) | 88 (27.5) |  | 46 (21.8) | 45 (21.3) |  |
| Plate/screw (n,%) | 28 (8.8) | 39 (12.2) |  | 22 (10.4) | 26 (12.3) |  |
| Multiple screws (n,%) | 104 (32.5) | 56 (17.5) |  | 54 (25.6) | 47 (22.3) |  |
| Bedridden time ≥ 5.0 days (n,%) | 134 (41.9) | 175 (54.7) | 0.258 | 100 (47.4) | 101 (47.9) | 0.009 |
| Operative blood loss ≥ 175 ml (n,%) | 101 (31.6) | 126 (39.4) | 0.164 | 71 (33.6) | 69 (32.7) | 0.020 |
| Surgery time ≥ 1.5hours (n,%) | 155 (48.4) | 144 (45.0) | 0.069 | 100 (47.4) | 97 (46.0) |  |
| Transfusion (n,%) | 19 (5.9) | 52 (16.3) | 0.332 | 17 (8.1) | 18 (8.5) | 0.017 |
| Postoperative ICU (n,%) | 4 (1.3) | 15 (4.7) | 0.203 | 4 (1.9) | 5 (2.4) | 0.033 |
| ASA |  |  |  |  |  |  |
| Ⅲ-Ⅴ(n,%) | 121 (37.8) | 172 (46.2) | 0.324 | 95 (45.0) | 97 (46.0) | 0.019 |
| Ⅰ-Ⅱ(n,%) | 199 (62.2) | 148 (53.8) |  | 116 (55.0) | 114 (54.0) |  |

**eTable8 Patient characteristics before and after propensity score matching by GAR(Q1 [< 0.137] vs. Q3[0.164-0.206])**

| Characteristics | Before matching | | | After matching | | |
| --- | --- | --- | --- | --- | --- | --- |
|  | Q1( < 0.137)  (n=320) | Q3(0.164-0.206) (n=320) | SMD | Q1( < 0.137)  (n=163) | Q3(0.164-0.206) (n=163) | SMD |
| Demographic |  |  |  |  |  |  |
| Age ≥75 (n,%) | 86 (26.9) | 193 (60.3) | 0.715 | 77 (47.2) | 84 (51.5) | 0.086 |
| Female gender (n,%) | 180 (56.3) | 202 (63.1) | 0.140 | 88 (54.0) | 101 (62.0) | 0.162 |
| Smoking (n,%) | 67 (20.9) | 47 (14.7) | 0.164 | 34 (20.9) | 28 (17.2) | 0.094 |
| Alcohol (n,%) | 36 (11.3) | 33 (10.3) | 0.030 | 20 (12.3) | 16 (9.8) | 0.078 |
| Comorbidities |  |  |  |  |  |  |
| Hypertension (n,%) | 98 (30.6) | 187 (58.4) | 0.582 | 83 (50.9) | 78 (47.9) | 0.061 |
| Diabetes (n,%) | 19 (5.9) | 64 (20.0) | 0.427 | 19 (11.7) | 21 (12.9) | 0.037 |
| COPD (n,%) | 21 (6.6) | 46 (14.4) | 0.257 | 16 (9.8) | 15 (9.2) | 0.021 |
| Cardiovascular (n,%) | 64 (20.0) | 109 (34.1) | 0.320 | 46 (28.2) | 45 (27.6) | 0.014 |
| Stroke (n,%) | 52 (16.3) | 83 (25.9) | 0.239 | 40 (24.5) | 39 (23.9) | 0.014 |
| Dementia (n,%) | 11 (3.4) | 14 (4.4) | 0.048 | 8 (4.9) | 8 (4.9) | <0.001 |
| Intracerebral hemorrhage (n,%) | 10 (3.1) | 21 (6.6) | 0.160 | 10 (5.1) | 6 (3.7) | 0.113 |
| Chronic liver disease (n,%) | 8 (2.5) | 11 (3.4) | 0.055 | 3 (1.8) | 3 (1.8) | <0.001 |
| Chronic kidney disease (n,%) | 10 (3.1) | 17 (5.3) | 0.109 | 7 (4.3) | 10 (6.1) | 0.083 |
| Rheumatoid arthritis (n,%) | 6 (1.9) | 5 (1.6) | 0.024 | 4 (2.5) | 2 (1.2) | 0.091 |
| Readmission (n,%) | 69 (21.6) | 97 (30.3) | 0.200 | 54 (33.1) | 50 (30.7) | 0.053 |
| Fracture type |  |  |  |  |  |  |
| Femoral neck fracture (n,%) | 228 (71.3) | 137 (42.8) | 0.482 | 92 (56.4) | 86 (52.8) | 0.049 |
| Intertrochanteric fracture (n,%) | 74 (23.1) | 166 (51.9) |  | 54 (33.1) | 71 (43.6) |  |
| Subtrochanteric fracture (n,%) | 18 (5.6) | 17 (5.3) |  | 17 (10.4) | 6 (3.7) |  |
| Surgery type |  |  |  |  |  |  |
| Total hip arthroplasty (n,%) | 62 (19.4) | 33 (10.3) | 0.196 | 25 (15.3) | 26 (16.0) | 0.088 |
| Hemiarthroplasty (n,%) | 64 (20.0) | 77 (24.1) |  | 44 (27.0) | 41 (25.2) |  |
| Intramedullary nail (n,%) | 62 (19.4) | 131 (40.9) |  | 43 (26.4) | 57 (35.0) |  |
| Plate/screw (n,%) | 28 (8.8) | 50 (15.6) |  | 24 (14.7) | 19 (11.7) |  |
| Multiple screws (n,%) | 104 (32.5) | 29 (9.1) |  | 27 (16.6) | 20 (12.3) |  |
| Bedridden time ≥ 5.0 days (n,%) | 134 (41.9) | 195 (60.9) | 0.388 | 85 (52.1) | 82 (50.3) | 0.037 |
| Operative blood loss ≥ 175 ml (n,%) | 101 (31.6) | 140 (43.8) | 0.253 | 59 (36.2) | 56 (34.4) | 0.038 |
| Surgery time ≥ 1.5 hours (n,%) | 155 (48.4) | 174 (54.4) | 0.196 | 89 (54.6) | 83 (50.9) | 0.074 |
| Transfusion (n,%) | 19 (5.9) | 66 (20.6) | 0.443 | 16 (9.8) | 18 (11.0) | 0.040 |
| Postoperative ICU (n,%) | 4 (1.3) | 21 (6.6) | 0.276 | 4 (2.5) | 0 (0) | 0.224 |
| ASA |  |  |  |  |  |  |
| Ⅲ-Ⅴ(n,%) | 121 (37.8) | 198 (61.9) | 0.495 | 89 (54.6) | 89 (54.6) | <0.001 |
| Ⅰ-Ⅱ(n,%) | 199 (62.2) | 122 (38.1) |  | 74 (45.4) | 74 (45.4) |  |

**eTable9 Patient characteristics before and after propensity score matching by GAR(Q1 [ < 0.137] vs. Q4[ ≥ 0.206])**

| Characteristics | Before matching | | | After matching | | |
| --- | --- | --- | --- | --- | --- | --- |
|  | Q1( < 0.137)  (n=743) | Q4( ≥ 0.206) (n=536) | SMD | Q1( < 0.137)  (n=349) | Q4( ≥ 0.206) (n=349) | SMD |
| Demographic |  |  |  |  |  |  |
| Age ≥75 (n,%) | 86 (26.9) | 219 (68.7) | 0.919 | 60 (57.7) | 63 (60.6) | 0.058 |
| Female gender (n,%) | 180 (56.3) | 212 (66.5) | 0.210 | 57 (54.8) | 60 (57.7) | 0.058 |
| Smoking (n,%) | 67 (20.9) | 41 (12.9) | 0.217 | 23 (22.1) | 19 (18.3) | 0.095 |
| Alcohol (n,%) | 36 (11.3) | 25 (7.8) | 0.116 | 10 (9.6) | 11 (10.6) | 0.032 |
| Comorbidities |  |  |  |  |  |  |
| Hypertension (n,%) | 98 (30.6) | 205 (64.3) | 0.714 | 54 (51.9) | 54 (51.9) | <0.001 |
| Diabetes (n,%) | 19 (5.9) | 184 (57.7) | 1.334 | 19 (18.3) | 21 (20.2) | 0.049 |
| COPD (n,%) | 21 (6.6) | 52 (16.3) | 0.309 | 10 (9.6) | 17 (16.3) | 0.200 |
| Cardiovascular (n,%) | 64 (20.0) | 128 (40.1) | 0.449 | 34 (32.7) | 38 (36.5) | 0.081 |
| Stroke (n,%) | 52 (16.3) | 120 (37.6) | 0.495 | 31 (29.8) | 28 (26.9) | 0.064 |
| Dementia (n,%) | 11 (3.4) | 12 (3.8) | 0.017 | 3 (2.9) | 3 (2.9) | <0.001 |
| Intracerebral hemorrhage (n,%) | 10 (3.1) | 23 (7.2) | 0.185 | 6 (5.8) | 7 (6.7) | 0.040 |
| Chronic liver disease (n,%) | 8 (2.5) | 25 (7.8) | 0.242 | 4 (3.8) | 5 (4.8) | 0.047 |
| Chronic kidney disease (n,%) | 10 (3.1) | 21 (6.6) | 0.161 | 6 (5.8) | 4 (3.8) | 0.090 |
| Rheumatoid arthritis (n,%) | 6 (1.9) | 5 (1.6) | 0.024 | 3 (2.9) | 2 (1.9) | 0.063 |
| Readmission (n,%) | 69 (21.6) | 117 (36.7) | 0.337 | 35 (33.7) | 35 (33.7) | <0.001 |
| Fracture type |  |  |  |  |  |  |
| Femoral neck fracture (n,%) | 228 (71.3) | 129 (40.4) | 0.552 | 49 (47.1) | 49 (47.1) | 0.073 |
| Intertrochanteric fracture (n,%) | 74 (23.1) | 165 (51.7) |  | 42 (40.4) | 47 (45.2) |  |
| Subtrochanteric fracture (n,%) | 18 (5.6) | 25 (7.8) |  | 13 (12.5) | 8 (7.7) |  |
| Surgery type |  |  |  |  |  |  |
| Total hip arthroplasty (n,%) | 62 (19.4) | 35 (11.0) | 0.242 | 13 (12.5) | 8 (7.7) | <0.001 |
| Hemiarthroplasty (n,%) | 64 (20.0) | 76 (23.8) |  | 27 (26.0) | 32 (30.8) |  |
| Intramedullary nail (n,%) | 62 (19.4) | 135 (42.3) |  | 34 (32.7) | 38 (36.5) |  |
| Plate/screw (n,%) | 28 (8.8) | 53 (16.6) |  | 18 (17.3) | 15 (14.4) |  |
| Multiple screws (n,%) | 104 (32.5) | 20 (6.3) |  | 12 (11.5) | 11 (10.6) |  |
| Bedridden time ≥ 5.0 days (n,%) | 134 (41.9) | 220 (69.0) | 0.566 | 61 (58.7) | 63 (60.6) | 0.039 |
| Operative blood loss ≥ 175 ml (n,%) | 101 (31.6) | 152 (47.6) | 0.333 | 45 (43.3) | 42 (40.4) | 0.058 |
| Surgery time ≥ 1.5 hours (n,%) | 155 (48.4) | 177 (55.5) | 0.141 | 55 (52.9) | 51 (49.0) | 0.077 |
| Transfusion (n,%) | 19 (5.9) | 73 (22.9) | 0.496 | 15 (14.4) | 15 (14.4) | <0.001 |
| Postoperative ICU (n,%) | 4 (1.3) | 26 (8.2) | 0.330 | 4 (3.8) | 2 (1.9) | 0.115 |
| ASA |  |  |  |  |  |  |
| Ⅲ-Ⅴ(n,%) | 121 (37.8) | 221 (69.3) | 0.664 | 65 (62.5) | 64 (61.5) | 0.020 |
| Ⅰ-Ⅱ(n,%) | 199 (62.2) | 98 (30.7) |  | 39 (37.5) | 40 (38.5) |  |
